# Haplotype-Resolved Chromosome-Scale Genome Assembly of *Lonicera macranthoides* Hand.-Mazz. Reveals Genetic Determinants of Bioactive Compound Biosynthesis and Phytohormone-Mediated Metabolic Regulation

**DOI:** 10.3390/biology15212023

**Published:** 2026-11-06

**Authors:** Qundong Liu, Xuhong Song, Tao Dou, Gang Ding, Longyun Li

**Affiliations:** 1 Chongqing Academy of Chinese Materia Medica, Chongqing 400065, China; lettuceliu@outlook.com (Q.L.); songxuhong@cqacmm.com (X.S.); doutao2018@126.com (T.D.); ppsad@outlook.com (G.D.); 2 China Academy of Chinese Medical Sciences, Beijing 100700, China

**Keywords:** *Lonicera macranthoides*, genome assembly, secondary metabolism, chlorogenic acid, triterpenoid biosynthesis

## Abstract

*Lonicera macranthoides* is a traditional Chinese medicinal herb valued for its chlorogenic acid (CGA) and triterpenoid saponins. To elucidate the genetic basis of these medicinal properties, we generated a haplotype-resolved chromosome-scale genome assembly combining PacBio long-read sequencing and High-throughput Chromosome Conformation Capture (Hi-C) data. The assembly comprises two haplomes, LmacA (857.17 Mb, contig N50 = 31.19 Mb) and LmacB (824.65 Mb, contig N50 = 27.17 Mb), representing the most contiguous genomic resource for the genus to date. Comparative genomic analyses identified species-specific gene families enriched in triterpenoid biosynthesis, phenylpropanoid metabolism, and stress-response pathways. Integrated GWAS, transcriptomic, and metabolomic analyses identified key genes regulating CGA and triterpenoid saponin biosynthesis, including a *UGT* cluster, an *HCT* cluster, and MYB transcription factors. We further found evidence that jasmonic acid and isopentenyl adenine dynamically balance floral organ development and secondary metabolite accumulation via MYB/bHLH transcription factors and MAPK signaling. This study establishes the first biosynthetic pathways for CGA and saponins in *L. macranthoides*, reveals regulatory mechanisms driven by haplotype structural variations (notably a Chr7 inversion), and provides genomic resources for molecular breeding and bioactive compound exploitation.

## Simple Summary

*Lonicera macranthoides* is a traditional Chinese medicinal plant. Its dried flower buds and flowers contain active compounds such as chlorogenic acid (CGA) and saponins, which are responsible for its medicinal properties. However, how these compounds are produced in the plant is not well understood. In this study, we built a high-quality genome map of *L. macranthoides*, separating it into two haplotype copies of 857 Mb and 825 Mb. We also studied three different types of the plant at three to five flower development stages. By combining genome-wide association (GWAS), gene expression, and metabolite data, we identified key genes that regulate the production of CGA and saponins. We further found that plant hormones help balance flower development and compound accumulation. This study provides a valuable resource for understanding and improving this medicinal plant.

## 1. Introduction

*Lonicera macranthoides* Hand.-Mazz. is a perennial medicinal plant whose flowers and flower buds have been used as Lonicerae Flos (known as “ShanYinHua” in Chinese) in Traditional Chinese Medicine for centuries [1,2]. Pharmacological studies have shown that Lonicerae Flos possesses anti-inflammatory, antimicrobial, antioxidant, hepatoprotective, and anticancer activities, which are mainly attributed to its secondary metabolites, including organic acids, flavonoids, iridoids, and triterpenoid saponins [3,4,5]. Among these, chlorogenic acids (CGAs) are the dominant bioactive compounds in *L. macranthoides*, with concentrations comparable to those in *Lonicera japonica* [4,6,7]. CGAs exhibit neuroprotective, anti-inflammatory, antioxidant, antimicrobial, cardioprotective, antidiabetic, hepatoprotective, and antitumor properties [8,9,10,11,12]. The CGA biosynthetic pathway has been extensively studied and involves three main routes with key enzymes such as hydroxycinnamoyl-CoA quinate transferase (HQT), hydroxycinnamoyl-CoA shikimate and quinate hydroxycinnamoyl transferase (HCT) and p-coumarate 3′-hydroxylase [5,13]. In *L. macranthoides*, CGA levels vary across tissues, with the highest concentration in young leaves, followed by young stems and mature flowers [6]. Transcriptomic and metabolomic analyses have identified candidate genes involved in CGA biosynthesis [7], and the R2R3-MYB transcription factor LmMYB15 has been reported to regulate CGA biosynthesis and phenylpropanoid metabolism [13].

In addition to organic acids, *L. macranthoides* accumulates significantly higher concentrations of triterpenoid saponins than other *Lonicera* species [5,14,15,16]. The characteristic saponins are mainly pentacyclic triterpenoids, with hederagenin-type saponins being the most abundant [14,17]. Notable examples include macranthoidin B, dipsacoside B, and macranthoidin A [17]. Dipsacoside B exhibits anti-inflammatory, antiviral, antioxidative, anti-apoptotic, hepatoprotective, and nephroprotective effects, while macranthoidin B shows potent antitumor activity [12,18,19,20,21,22,23,24,25]. The biosynthesis of hederagenin-based saponins involves three main stages: formation of terpenoid precursors via the mevalonate pathway, cyclization to form the triterpenoid scaffold, and structural modification by cytochrome P450 enzymes and UDP-glycosyltransferases [5,17]. Although the general pathways are well-established, the biosynthetic pathways and regulatory mechanisms specific to *L. macranthoides* remain largely unknown [17,26].

**Figure 1:**
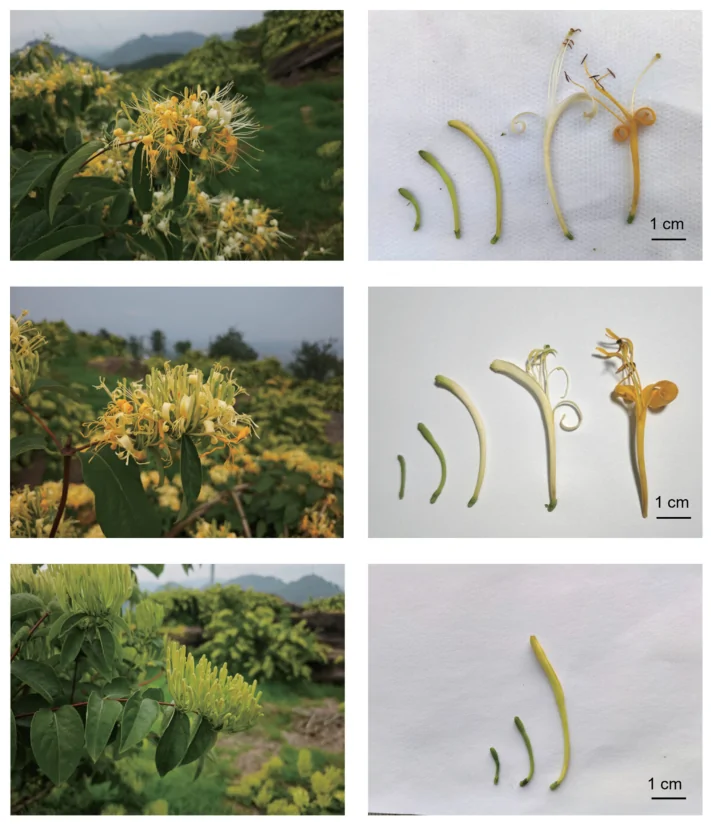
Three cultivars of *L. macranthoides*. From top to bottom: Wild Floriferous type (W), Transitional type (T), and Closed bud type (C). The five flowering stages of W and T type, and the three flowering stages of C type are shown on the right part.

Flower development stage is an important factor influencing the quality of medicinal ingredients in *L. macranthoides* [26,27,28]. The flowering process has been categorized into distinct stages, including: Bud (small, pale green buds), Green (green, rod-shaped buds), White (white, rod-shaped buds), Silver (white, dehiscent flowers), and Yellow (yellow, open flowers) (Figure 1) [29]. Germplasm variation also exists among cultivars. In our previous report, open wild-type buds of *L. macranthoides* showed higher CGA and saponin content than the unopened type [30]. In the current study, three cultivars were introduced: Wild floriferous type (W), Closed bud type (C) and Transitional type (T). The transitional type (T) is an intermediate phenotype that displays both flowering and closed-bud traits [26]. To our knowledge, no specific phylogenetic study has yet resolved the relationships among these three cultivars; they are considered intraspecific variants of *L. macranthoides* rather than distinct lineages [30]. The closed bud type is popular in cultivation due to high production and convenient harvesting, but has low CGA content and disease resistance [26,27]. The wild floriferous type has high genetic diversity but poor industrial production of flower buds due to drastic morphological changes (e.g., early corolla opening), which reduce the yield of harvestable buds [31,32]. Therefore, understanding the metabolite changes and regulatory mechanisms during flower bud splitting is necessary for breeding new cultivars with high bioactive compound content and disease resistance [26,27,33,34,35,36].

Genomic studies of *L. macranthoides* hold promise for elucidating phytochemical diversity, biosynthetic pathways, stress adaptation, and evolutionary history [26,37]. Although the nuclear and chloroplast genomes of *L. macranthoides* have been assembled [17,38], a haplotype-resolved genome assembly has not yet been reported despite the species being diploid. The differential expression of haplotypes, their asymmetric evolution, and their roles in metabolite accumulation remain unexplored.

In this study, we report a high-quality, haplotype-resolved chromosome-level genome assembly of *L. macranthoides*. Integrated metabolomic, transcriptomic, and Genome-wide association studies (GWAS) analyses identified key enzymes involved in CGA and triterpenoid saponin biosynthesis, and transcriptomic analysis of hormone signaling provided insights into flower development regulation. This genomic resource will facilitate further molecular and pharmacological studies of this medicinal species.

## 2. Materials and Methods

### 2.1. Plant Materials for Genome Sequencing

Seedlings of *L. macranthoides* were cultivated in Suiyang, Zunyi City, Guizhou Province (27.93° N, 107.39° E). Genomic DNA was extracted from the leaves of an individual plant of the W (wild cultivar) type using a modified CTAB method [39]. DNA sequencing was performed on the MGISEQ-2000 (MGI, Shenzhen, China) platform using 150 bp paired-end mode, and raw reads were filtered by fastp [40] (version: 0.21.0, parameters: default parameters). PCR-free SMRT bell libraries were constructed and sequenced on the PacBio Sequel II platform (PacBio, Menlo Park, CA, USA). Filtered subreads were processed to generate HiFi reads using SMRTLink v11.0 (parameters: -min-passes = 3, -min-rq = 0.99). Hi-C library was constructed by the Wuhan Benagen Technology Co., Ltd. (Wuhan, China) and sequenced on the Illumina NovaSeq6000 system (Illumina, San Diego, CA, USA) with a read length of 150 bp.

#### Library Construction for Genome Sequencing

For MGISEQ-2000 short-read sequencing, genomic DNA was fragmented to the appropriate size, end-repaired, A-tailed, and ligated to sequencing adapters using the MGIEasy DNA Library Prep Kit (MGI, Shenzhen, China) following the manufacturer’s instructions. The library was quantified, quality-controlled, and sequenced on the MGISEQ-2000 platform (MGI, Shenzhen, China) in 150 bp paired-end mode.

For PacBio HiFi sequencing, high-molecular-weight genomic DNA was used to construct a PCR-free SMRTbell library. Briefly, DNA was sheared to the target size, end-repaired, and ligated to SMRTbell adapters provided in the SMRTbell Express Template Prep Kit 2.0 purchased from Pacific Biosciences (PacBio, Menlo Park, CA, USA). The SMRTbell library was purified, quality-controlled, and sequenced on the PacBio Sequel II platform. Raw subreads were processed into HiFi reads using SMRTLink v11.0 with parameters -min-passes = 3 and -min-rq = 0.99.

For Hi-C sequencing, a Hi-C library was constructed by Wuhan Benagen Technology Co., Ltd. (Wuhan, China) using a standard Hi-C protocol. In brief, nuclei were cross-linked with 1% formaldehyde at room temperature for 10 min, and the reaction was quenched with 0.125 M glycine. The cross-linked chromatin was digested with DpnII (New England Biolabs, Ipswich, MA, USA) at 37 °C overnight. The digested ends were filled in with biotin-14-dATP using DNA polymerase I (Klenow fragment), and proximity ligation was performed with T4 DNA ligase at 16 °C for 4 h. After ligation, the cross-links were reversed by incubation with proteinase K at 65 °C overnight. The purified DNA was then fragmented, biotin-enriched, and converted into an Illumina sequencing library. The final library was sequenced on the Illumina NovaSeq 6000 platform (Illumina, San Diego, CA, USA) with 150 bp paired-end reads.

### 2.2. K-mer Analysis and Genome Assembly

MGISEQ-2000 short reads were used for genome survey analysis. K-mer analysis was performed with Jellyfish (v2.2.10) [41] using default parameters and a k-mer size of 19. Based on the resulting k-mer frequency and depth distribution, genome size was estimated using GCE (version 1.1.0) with default parameters [42].

PacBio HiFi reads were used for primary contig assembly. We used Hifiasm [43] (version 0.16.1) with -t 64 to assemble the initial contigs. Hifiasm loads all reads into memory, and the reads are aligned all-vs-all to find the overlap between reads. Three rounds of self-correction for reads with overlaps were performed by Hifiasm by default. All-vs-all comparison was performed again on the error-corrected reads, and the overlap between reads was used to construct a string graph. Haplotype construction was performed with the help of reads with differences in base types at individual positions. The N50 size of the genome was used to evaluate the continuity of the genome. The consistency of the genome was evaluated by calculating the second-generation alignment rate and coverage using BWA [44] (version: 0.7.17, parameter: mem). MGISEQ-2000 short reads were used for read mapping during the analysis. Genome completeness was assessed using BUSCO (Benchmarking Universal Single-Copy Orthologs) v5.3.0 [45] with the embryophyta_odb10 lineage dataset in protein mode (*e*-value threshold: 1 × 10^−5^).

### 2.3. Haplotype-Resolved Genome Assembly

Hi-C reads were used for chromosome-level scaffolding. Two haplotypes were assembled by Hifiasm (version 0.16.1, -t 64) and processed separately. Each haplotype assembly was scaffolded separately with Hi-C data before chromosome construction. Contigs were assigned to haplotypes based on Hi-C interaction patterns and allele-specific k-mer differences identified by Hifiasm. The amount of effective Hi-C data was evaluated using Juicer [46] (version: 1.6, parameters: default), and then clustered into chromosomes by 3D-DNA (version: 180419, parameters: -m diploid). Misordered and misoriented contigs were corrected using Juicebox (version: 1.11.08). During subsequent manual curation, only sequences identified as clear misassemblies or duplicated haplotypes with identical sequences were removed, while haplotype-specific regions were carefully retained to obtain the final genome sequences. A heatmap of chromosome Hi-C interactions was plotted based on the contig interaction strength and position by HiCExplorer [47] (version: 3.6, parameters: default). Contiguity, consistency, and completeness were used to evaluate the genome assembly quality. Genomic collinearity analysis for two haplotypes was performed using MUMmer [48] (version: 4.0.0rc1, parameters: -mum -c 90 -l 5000).

K-mer-based completeness of each haplotype assembly was evaluated with Merqury [49] (https://github.com/marbl/merqury, version: 1.3, accessed on 29 August 2026) using default parameters. Switch error rates were estimated by aligning the phased haplotype assemblies to the PacBio HiFi reads and comparing the phasing of adjacent heterozygous sites. A switch error was defined as an incorrectly phased pair of adjacent heterozygous SNPs, and the switch error rate was calculated as the number of switch errors divided by the total number of adjacent heterozygous SNP pairs. The resulting switch error rates were 4.72% for LmacA and 5.08% for LmacB, respectively.

### 2.4. Genome Annotation

Genome annotation includes repetitive sequence annotation, gene annotation (gene structure prediction and gene function annotation), and non-coding RNA annotation. Genome annotation was performed separately for each haplotype (LmacA and LmacB) using the same pipeline. *De novo* repeat identification was performed separately for each haplotype (LmacA and LmacB) using the same pipeline. For each haplotype, RepeatModeler [50] (version: 2.0.4) was run with default parameters to generate a raw repeat model library. In parallel, LTR retrotransposons were predicted using LTR_FINDER_parallel (parameters: -threads 16 -harvest_out -size 1,000,000 -time 300) and LTRharvest [51] (version: 1.62, parameters: -minlenltr 100 -maxlenltr 7000 -mintsd 4 -maxtsd 6 -motif TGCA -motifmis 1 -similar 85 -vic 10 -seed 20 -seqids yes). The LTR predictions from both tools were combined and filtered for redundancy using LTR_retriever [52] (version: 2.9.0, -threads 16 -noanno), yielding a non-redundant LTR library. The raw RepeatModeler library and the non-redundant LTR library were then merged directly by concatenation to generate the *de novo* repeat library. This *de novo* repeat library represents the set of repeat families predicted ab initio from the *L. macranthoides* genome itself. No additional inter-library redundancy removal was performed at this stage. Unclassified repeat sequences in the *de novo* library were classified using TEclass [53] (version: 2.1.3, default parameters).

For homology-based annotation, the curated RepBase library (version: 21.05) was combined with the *de novo* library to construct the final repeat database. RepeatMasker [54] (v4.1.5, parameters: -noLowSimple -*p* value 0.0001) was used to identify and mask repetitive elements through nucleotide-level similarity searches against this combined database; the resulting genome was hard-masked (repeat sequences replaced with N) for downstream gene prediction. RepeatProteinMask (version: 4.1.5, parameters: -noLowSimple -*p* value 0.0001) was employed to detect repetitive elements at the protein level by searching against the RepeatPeps database (RepBase protein database, version: 21.05). Tandem repeats were identified using TRF (version: 4.09, parameters: match = 2, mismatch = 7, delta = 7, PM = 80, PI = 10, minscore = 50, maxperiod = 2000, with -d -h) and MISA (version: 2.1, with default parameters: mono- ≥ 10, di- ≥ 6, tri- ≥ 5, tetra- ≥5, penta- ≥ 5, hexa- ≥ 5). Finally, all repeat predictions (*de novo* + RepBase, TE proteins, and tandem repeats) were merged, and overlapping or redundant annotations were resolved to produce the final combined TE set for each haplotype.

Transcriptome reconstruction, homology prediction and ab initio prediction were integrated to annotate gene models. For second-generation transcriptome reconstruction, raw RNA-seq reads were filtered using Fastp (version: 0.23.2, parameter: -L -w 16). Filtered reads were aligned to the genome with Hisat2 (version: 2.2.1, parameter: -dta -p 16), and transcripts were reconstructed using StringTie (version: 2.2.1, parameter: -p 16). For third-generation transcriptome reconstruction, PacBio Iso-Seq data (hereafter referred to as PacBio HiFi transcript reads) were used. Raw subreads were processed to generate full-length non-concatemer (FLNC) reads using the standard Iso-Seq pipeline (SMRT Link, version: 12.0). The FLNC reads were then aligned to the genome in splice-aware mode with Minimap2 (version: 2.26-r1175, parameters: -t 16 -ax splice -uf -secondary = no). Transcript models were reconstructed and collapsed using cDNA-Cupcake (version: 1.0, default parameters) and merged with TAMA (version:1.0, parameters: -f filelist.txt -p merge). Finally, coding sequences were predicted with TransDecoder (version: 5.7.0; TransDecoder. LongOrfs -m 50, TransDecoder. Predict -retain_long_orfs_length 150). For homology prediction, the protein sequences of five closely related species were used: *Arabidopsis thaliana*, *L. japonica*, *Daucus carota*, *Solanum lycopersicum*, and *Oryza sativa*. These proteins were aligned to the genome using TBLASTN (version: 2.13.0, default parameters), and candidate gene structures were refined with Exonerate (version: 2.4.0, -model protein2genome -showtargetgff 1). For ab initio prediction, Augustus (version: 3.2.1) was used to train a coding gene model. All gene prediction data were finally combined into a consensus gene set using the MAKER pipeline (version: 3.31.8). The completeness of the genome annotation was assessed using the BUSCO software (version: v5.4.7, parameters: usco -m prot -c 16). The annotation of gene functions was performed using Diamond, InterProScan, Hmmscan, Kofam and Pfam to predict motifs, domains, protein functions, and metabolic pathways of these genes.

Noncoding RNA annotation was performed separately for each haplotype (LmacA and LmacB) using the same pipeline. tRNA genes were predicted using tRNAscan-SE (version: 2.0.12, parameters: -E -j tRNA.gff -o tRNA.result -f tRNA.struct -thread 16). rRNA genes were predicted using RNAmmer (version: 1.2, parameters: -S euk -m tsu,lsu,ssu). Other structured noncoding RNAs (e.g., snRNAs, miRNAs) were identified by searching against the Rfam covariance model database (version: 14.9, file: Rfam.cm) using INFERNAL (version: 1.1.4). Specifically, the cmscan program was used with the parameters: --cut_ga --rfam --nohmmonly --fmt 2. The -Z option was set to twice the genome length for each haplotype (actual values: 1,714,349,310 for LmacA and 1,649,293,700 for LmacB). The Rfam.cm file was downloaded from the Rfam website and compressed with cmpress before running cmscan. Clan information was supplied via --clanin Rfam.clanin to manage overlapping family hits. In addition to the standard output, tabular results were generated using the --tblout parameter. To reconcile overlapping predictions, priority was given to tRNA and rRNA annotations from tRNAscan-SE (version: 2.0.12) and RNAmmer (version: 1.2), respectively; any INFERNAL hits overlapping these regions were removed. This approach identifies homologs of known structured RNA families rather than providing a comprehensive prediction of all ncRNAs.

### 2.5. Evolutionary Analyses

Identification of homologous genes is important for evolutionary analysis. To explore the evolution of the *L. macranthoides* genome, we performed comparative genomics analyses with 13 additional plant species, resulting in a total of 14 species included in the phylogeny. The additional species were: *L. japonica*, *Lonicera maackii*, *Lonicera caerulea*, *Panax stipuleanatus*, *Apium graveolens*, *Daucus carota*, *Platycodon grandiflorus*, *Chrysanthemum nankingense*, *Lactuca sativa*, *Arctium lappa*, *Ilex polyneura*, *Ilex latifolia*, *and Camellia sinensis*. Genome/proteome sources and version numbers for all species are provided in Supplementary Table S1. The comparison was conducted using Blastp (version: 2.6.0; parameters: -*e* value 1 × 10^−5^ -outfmt 6). Gene families were defined as orthogroups inferred by OrthoFinder (version: 2.3.12; parameters: -M ms), which clusters genes from all species into groups based on sequence similarity using the MCL algorithm (default inflation parameter 1.5). An orthogroup represents a gene family that includes homologous genes from different species and may contain multiple paralogs per species. Single-copy orthologs were defined as orthogroups containing exactly one gene per species. To avoid haplotype copies being counted as gene-family duplications, only a single representative gene set from haplotype LmacA was used for *L. macranthoides*. Before orthogroup clustering, alternative transcript isoforms were filtered by retaining only the longest transcript per gene. GO and KEGG annotation of shared and unique genes using the R package clusterProfiler (version 3.18.1).

A total of 1346 single-copy orthogroups were identified across the 14 species and used for phylogenetic reconstruction. Protein sequences of each single-copy orthogroup were aligned using MAFFT (version: 7.471) with the L-INS-i algorithm, and ambiguously aligned regions were trimmed using TrimAl (version: 1.2rev59, -gt 0.2). The filtered alignments were then concatenated into a single supermatrix. Phylogenetic relationships were inferred with RAxML (version: 8.2.10) using the PROTGAMMAAUTO substitution model (best-fit model automatically selected by RAxML based on the data; cross-checked with ModelTest-NG). Node support was assessed with 100 rapid bootstrap replicates followed by a thorough maximum-likelihood search (-f a option). *Camellia sinensis* was designated as the outgroup.

Divergence times were estimated using MCMCTREE in the PAML package (version 4.9) with a correlated molecular clock model. Calibration nodes were obtained from the TimeTree database (http://www.timetree.org/, accessed on 10 August 2025) and included the following: *C. sinensis* and *I. latifolia*: 101.5–115.6 MYA, *L. japonica* and *L. sativa*: 76.0–97.4 MYA, D. carota and P. stipuleanatus: 54.3–69.0 MYA. Two independent MCMC chains were run for 1,000,000 generations, with the first 10% discarded as burn-in. Convergence was assessed by ensuring an effective sample size (ESS) > 200 for all parameters. CAFE (version 3.1) was used to estimate ancestral gene family sizes and to infer gene family contractions and expansions along each branch of the species tree using a birth–death model. The gene family birth–death rate (λ) was estimated by maximum likelihood from the phylogenetic tree and observed gene family sizes. An assembly/annotation error model was applied with an error probability (eP = 0.05). Gene families containing more than 100 genes were filtered out prior to analysis. The same representative gene set (LmacA) was used as input for CAFE. Gene families with *p* ≤ 0.05 were considered significantly expanded or contracted. For GO and KEGG enrichment analyses, the test gene sets were the genes from significantly expanded and contracted families, and the background gene set comprised all annotated genes from the LmacA representative gene set. GO annotation used database version 2024-01, and KEGG used release version 104.0. Multiple-testing correction was performed using the Benjamini–Hochberg false discovery rate (FDR) method.

Whole-genome duplication (WGD) events in the *L. macranthoides* genome were identified using two independent approaches. First, the 4DTV (four-fold degenerate transversion) method implemented in ParaAT (version 2.0, default parameters) was used to calculate 4DTV values for paralogous gene pairs. Second, the Ks-based WGD detection pipeline implemented in ksrates (version 2.0.0, default parameters) was applied; ksrates internally detects paralogous and orthologous gene pairs and estimates Ks values using its built-in workflow. No additional MCScanX- or PAML-based Ks distribution analysis was performed.

To detect positive selection in the *L. macranthoides* genome, protein sequences of each single-copy gene family were aligned using MAFFT (parameters: -localpair -maxiterate 1000) and converted to codon alignments using PAL2NAL (version: 14). The foreground lineage was set as *L. macranthoides*, with all other species as the background.

Positive selection was tested using the branch-site model implemented in CODEML from PAML (v4.9). In this analysis, model A, which allows sites under positive selection (ω>1) on the foreground branch, was used as the alternative model, whereas the corresponding null model constrained ω=1 on the foreground branch. Likelihood ratio tests (LRTs) were performed using the Chi2 program. Multiple-testing correction was applied using the Benjamini–Hochberg false discovery rate (FDR) method. Positively selected sites were identified using the Bayes empirical Bayes (BEB) approach with a posterior probability threshold > 0.95.

With haplotype A as the reference, whole-genome alignment of haplotype genomes was performed with MUMmer (version: 4.0.0rc1, parameters: default), followed by detection of structural variants with Synteny and Rearrangement Identifier (SyRI) (version: 1.5, parameters: default).

### 2.6. Plant Materials for GWAS and Metabolite Analyses

A total of 102 *L. macranthoides* plants were collected from Suiyang, Zunyi City, Guizhou Province (27.93° N, 107.39° E), China. The population comprised mixed floral types, including W, T, and C types. Flower buds were harvested at the White stage (the last bud stage before flowering). For each plant, flower buds were collected under the same sampling conditions and used for both DNA extraction (for sequencing) and metabolite measurement. Genomic DNA was extracted from each of the 102 plants and subjected to whole-genome resequencing on the MGISEQ-2000 platform (MGI, Shenzhen, China) using 150 bp paired-end reads. The mean sequencing depth was approximately 12× per sample. The concentrations of chlorogenic acid (CGA) and Dipsacoside B were determined using HPLC, as described in our previous study [30].

### 2.7. GWAS Analysis

Phenotypic data (CGA and Dipsacoside B concentrations) were assessed for normal distribution and correlation using the R package PerformanceAnalytics (version: 2.0.4). CGA and Dipsacoside B concentrations were measured by HPLC as previously described [30]. Each sample was measured in three technical replicates, and the mean value was used for GWAS. Raw sequencing data were quality-controlled and filtered with fastp (version: 0.23.4; parameters: -3 -5 -e 20 -l 50 -detect_adapter_for_pe) to obtain clean reads. Clean reads were aligned to the LmacA haplotype assembly using bwa-mem2 (version: 2.2.1; parameters: mem). To assess potential mapping bias, reads were also aligned to the LmacB haplotype assembly as an alternative reference, and variants were cross-validated between the two mappings; only consistent calls were retained. Variant detection was performed using GATK (version: 4.5.0.0; HaplotypeCaller, CombineGVCFs, GenotypeGVCFs), and SNPs and INDELs were identified and annotated with ANNOVAR (version: 20200607; convert2annovar.pl, annotate_variation.pl). Genotype data were filtered with VCFtools (version: 0.1.16; parameters: --min-alleles 2 --max-alleles 2 --maf 0.05 --max-missing 0.8) to retain biallelic sites with MAF ≥ 0.05 and ≤20% missing genotypes. After quality filtering, 21,225,621 SNPs were retained for association testing.

For each phenotype, association analyses were conducted using GEMMA (version: 0.98.5; lm and lmm), EMMAX (version: beta-07Mar2010; Efficient Mixed-Model), and rMVP (version: 1.0.4; GLM, MLM, and FarmCPU), incorporating the first three principal components (PCs) from PCA as covariates. A centered IBS kinship matrix was used to control for population structure. Considering the extent of linkage disequilibrium in this population (r^2^ ≈ 0.5 at 50 kb), the effective number of independent markers is far smaller than the total number of SNPs; a genome-wide significance threshold of *p* < 5 × 10^−8^ was therefore applied, which is conservative relative to the Bonferroni threshold corresponding to the effective number of independent markers. Loci reaching this threshold in at least one model were retained. Based on the significant loci, regions extending ±50 kb upstream and downstream were subjected to linkage disequilibrium (LD) block identification. The ±50 kb interval was chosen based on the observed LD decay in this population, where r^2^ declined to approximately 0.5 at 50 kb. Candidate genes at each significant locus were defined as the annotated genes nearest to the peak SNP (the closest upstream and downstream genes). Where the nearest genes formed part of a tandem gene cluster functionally related to the target compound, the entire cluster was considered as the candidate region; the ±50 kb window was used for LD block identification and haplotype analysis, not as a strict cutoff for candidate gene selection. Samples were then categorized into distinct genotype groups at the significant loci, and phenotypic distributions were compared among groups, integrating variant annotation for contextual analysis. Note on structural variants: The major structural variant between LmacA and LmacB, particularly the Chr7 inversion, was not directly genotyped in the 102 plants. Therefore, the associations reported here support nearby SNPs within or near the inversion region rather than the structural variant itself.

### 2.8. Transcriptome Analysis

Three cultivars (designated W, C, and T) were examined for flower developmental stages. For cultivars W and T, five flowering stages (Bud, Green, White, Silver, and Yellow) were sampled, whereas for cultivar C, only three stages (Bud, Green, White) were collected. At each stage and for each cultivar, four biological replicates were obtained, with each replicate consisting of an individual plant. Therefore, a total of (5 + 5 + 3) × 4 = 52 RNA-seq libraries were constructed. All samples were immediately frozen in liquid nitrogen and stored until processing. Total RNA was extracted from each individual replicate using the R6827 Plant RNA Kit, and transcriptome libraries were prepared using the MGI Easy RNA Library Prep Kit for BGI (MGI Tech, Shenzhen, China) according to the manufacturer’s protocol. Briefly, poly(A) mRNA was enriched from total RNA using oligo(dT)-conjugated magnetic beads and then fragmented into short fragments. First-strand cDNA was synthesized using random primers and reverse transcriptase, followed by second-strand cDNA synthesis to generate double-stranded cDNA. The resulting cDNA fragments underwent end repair, 3′ adenylation, and ligation with BGI-specific adapters. The adapter-ligated products were amplified by PCR to construct the final sequencing libraries. Subsequently, the libraries were circularized to form single-stranded circular DNA, and the un-circularized linear DNA was digested. The circular DNA was then amplified via rolling circle amplification to generate DNA nanoballs (DNBs). Finally, the DNBs were sequenced on the DNBSEQ-T7 platform (MGI, Shenzhen, China)to generate paired-end reads. Total RNA integrity was assessed by 1% agarose gel electrophoresis. The integrity was judged by the presence of clear 28S and 18S rRNA bands, with an approximate 28S:18S intensity ratio of 2:1 and no obvious degradation smear. Samples with intact rRNA bands were used for library construction.

Raw FASTQ reads were processed with fastp (version 0.21.0) using default parameters to obtain high-quality clean reads for downstream analyses. The filtered transcriptome sequences were aligned to the reference genome using STAR [55] (version: 2.7.9a; parameters: default). Gene- and transcript-level expression was quantified using RSEM (version: 1.3.1) [56] with default parameters. The expected count matrix generated by RSEM was directly used as input for differential expression analysis with DESeq2 (version: 1.26.0) [57]. The DESeq2 design formula was set as cultivar + stage. Pairwise comparisons were performed to identify differentially expressed genes between cultivars at each stage and between stages within each cultivar. Genes with an adjusted *p*-value < 0.05 and |log2FoldChange| > 1 were considered significantly differentially expressed. GO and KEGG enrichment analysis was performed using the R clusterProfiler package (version: 3.18.1) [58].

### 2.9. Non-Targeted Metabolomics by Liquid Chromatography-Mass Spectrometry

Six biological replicates were performed per group following the same group strategy as RNA-Seq for LC-MS. Plant samples (20 mg ± 1 mg) were lyophilized, mixed with beads and 1000 μL extraction solution (MeOH: ACN: H_2_O, 2:2:1, *v*/*v*/*v*) containing deuterated internal standards, vortexed for 30 s, homogenized (35 Hz, 4 min), and sonicated for 5 min in a 4 °C water bath; this step was repeated three times. After incubation at −40 °C for 1 h, samples were centrifuged (12,000 rpm, 13,800× *g*, 15 min, 4 °C). The supernatant was transferred to a fresh glass vial. QC samples were prepared by mixing equal aliquots of supernatant from all samples. Each biological replicate was a single plant and was processed separately. LC-MS/MS was performed on a Vanquish UHPLC (Thermo Fisher Scientific, Waltham, MA, USA) coupled to an Orbitrap Exploris 120 mass spectrometer (Thermo Fisher Scientific, Waltham, MA, USA) with a Waters ACQUITY UPLC BEH Amide column (2.1 mm × 50 mm, 1.7 μm, Waters, Milford, MA, USA). Mobile phase A: 25 mM ammonium acetate (Cat. No.**73594**, Merck KGaA, Darmstadt, Germany) and 25 mM ammonia hydroxide (Cat. No.**5.33003.0050**, Merck KGaA, Darmstadt, Germany) in water (pH 9.75); mobile phase B: acetonitrile (Cat. No.**51101**, Thermo Fisher Scientific, Waltham, MA, USA). The gradient was as follows:0 –1 min, 95% B; 1–7 min, 95%→65% B; 7–8 min, 65%→40% B; 8–8.5 min, 40%→95% B; 8.5–10 min, 95% B. The flow rate was 0.4 mL/min, the column temperature was 40 °C, the auto sampler (Thermo Fisher Scientific, Waltham, MA, USA) was kept at 4 °C, and the injection volume was 2 μL. Full scan MS spectra were acquired over *m*/*z* 50–1000, with a run time of 10 min. The injection order was: blank → QC → samples; a pooled QC was injected every 10 samples, and a blank every 10 samples. The ESI source conditions were: sheath gas 50 Arb, aux gas 15 Arb, capillary temperature 320 °C, full MS resolution 60,000, MS/MS resolution 15,000, collision energy SNCE 20/30/40, spray voltage 3.8 kV (positive) or −3.4 kV (negative). MS/MS spectra were acquired in IDA mode under Xcalibur (version: 4.4, Thermo).

Raw mass spectrometry data were converted to mzXML format using ProteoWizard (version 3.0.24172), and all raw data were processed with the XCMS package (version: 3.6.1; https://bioconductor.org/packages/xcms/, accessed on 20 May 2024) in R for peak deconvolution, alignment, and integration. Peak detection used the centWave method with ppm = 20, peak width = c(6, 48), snthresh = 5, prefilter = c(5, 10). Alignment used obiwarp with profStep = 1. Features with RSD > 30% in QC were excluded; normalization was performed using QC based normalization; missing values were replaced by half of the minimum detected value. Metabolite annotation was performed against BiotreeDB (version: 3.0) with a mass tolerance of <10 ppm, retention time tolerance of 0.2 min, and MS/MS match score > 0.8. Authentic standards were used when available (MSI Level 1); Otherwise annotations were reported as MSI Level 2 or 3.

### 2.10. Construction of the Phytohormone-Mediated Regulatory Network

To resolve the hierarchical regulatory network governing corolla dehiscence, a time-ordered gene co-expression network (TO-GCN) was constructed using transcriptomic and targeted phytohormone metabolomic data as previously described [59]. Briefly, DEGs associated with hormone metabolism, signal transduction, and floral development were selected as structural genes. Phytohormones were treated as phenotypic nodes. The network edges were determined based on significant correlations (Pearson correlation) and time-lagged expression patterns between structural genes and TFs, as well as between TFs and hormones. The final network was visualized using Cytoscape (version 3.9.0) [60].

### 2.11. qRT-PCR Validation

Three biological replicates were performed per group, following the same grouping strategy as RNA-Seq. Each biological replicate was a single plant and was processed separately. Total RNA extraction, genomic DNA removal, and first-strand cDNA synthesis were performed as reported earlier [61] using the Vazyme fluorescent quantitative kit (ChamQ Universal SYBR qPCR Master Mix, Nanjing, China). Primers were designed using Primer3 (Table S2). qPCR was performed with three technical replicates per biological replicate under the following conditions: 95 °C for 30 s; 40 cycles of 95 °C for 10 s and 60 °C for 30 s, with a reaction volume of 20 μL. Product specificity was confirmed by melting-curve analysis showing a single peak, with no non-specific amplification or primer-dimers. No-template and no-RT controls were included. LguiScaf91G00363370 (an ATP synthase F1 subunit alphagene, named FR870019) was used as the internal control; its stable expression across all cultivars and floral stages was validated by geNorm (M < 0.5) using RNA-Seq data. Relative expression was calculated using the 2^−ΔΔCt^ method [62,63]. Statistical analysis was performed using one-way ANOVA with Tukey’s test. Information on all bioinformatics tools used in this study is provided in Table S3.

## 3. Results

### 3.1. Sequencing, Assembly and Annotation of the L. macranthoides Genome

**Figure 2:**
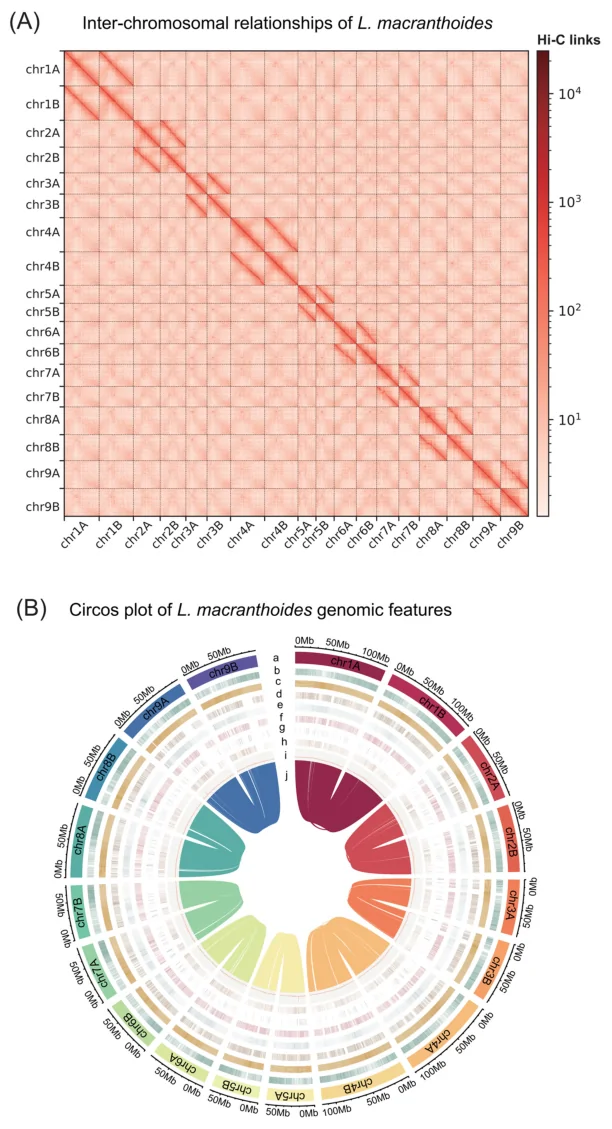
Genome assembly of *L. macranthoides*. (**A**) The HI-C interaction heatmaps showing the 18 pseudochromosomes of the two haplotypes. Heatmap colors show Hi-C links. (**B**) Circos plot of *L. macranthoides* genomic features. a, Circular representation of the 18 pseudochromosomes. b, gene density (10 Mb window). c, percentage of repeats (10 Mb window). d–h, structural variations between LmacA and LmacB haplomes, including deletions, duplications, insertions, inversions and translocations. i, GC content (10 Mb window). j, syntenic relationship between LmacA and LmacB chromosomes.

To uncover the genetic foundation of *L. macranthoides*, we produced a chromosome-scale, high-quality genome assembly. Genome size estimation via k-mer analysis revealed an 804.88 Mb genome with 1.36% heterozygosity and 49.67% repetitive sequences (Figure S1). Whole-genome sequencing yielded 88.09 Gb of BGI data (109.4× coverage), 66.48 Gb of HiFi data (82.6× coverage), and 76.43 Gb of Hi-C data (95.0× coverage) (Table S4). *De novo* assembly of contigs resolved two phased haplotypes: genome-level haplotype A (LmacA) and haplotype B (LmacB). Integration with a Hi-C-based physical map assigned 97.55% of sequences to 18 pseudochromosomes (Figure 2A,B; chromosome-specific statistics in Table S5). The final LmacA assembly spanned 857.17 Mb with a contig N50 of 31.19 Mb and 98.90% BUSCO completeness, while LmacB spanned 824.65 Mb with a contig N50 of 27.17 Mb and 99.10% BUSCO completeness (Table 1). The k-mer completeness values for the two haplotypes were 80.36% and 79.37%, respectively, as determined by Merqury. The switch error rates were 4.72% for LmacA and 5.08% for LmacB. These low values indicate that more than 95% of adjacent heterozygous sites were phased correctly in both haplotypes, demonstrating high phasing accuracy of the haplotype-resolved assemblies. No average across the two haplotypes was calculated; the two values are reported separately for each haplotype.

By integrating transcriptome, homolog-based searches, and ab initio prediction, we identified 36,792 protein-coding genes in LmacA (mean gene length: 5831 bp; BUSCO completeness: 99.0%) and 36,433 genes in LmacB (mean gene length: 5954 bp; BUSCO completeness: 99.0%) (Figure 2B, Table 2). Comparative analysis of gene length, CDS length, exon length, and intron length distributions revealed conservation with related species (Figure S2), supporting annotation quality. Although the mean intron lengths (1054.87 bp for LmacA and 1080.01 bp for LmacB) were substantially longer than those of species with compact genomes, their median values were only 426 bp and 434 bp, respectively. This discrepancy is driven by a long-tail effect: introns containing transposable elements (TEs) have a mean length of 1771 bp, whereas TE-free introns average only 265 bp (Figure S2). Repetitive elements occupied 65.38% (560,438,594 bp) of the LmacA genome and 65.28% (538,357,766 bp) of LmacB. This annotation-based repeat content is higher than the preliminary k-mer-based estimate (49.67%), as k-mer frequency analysis tends to underestimate highly divergent or complex repeats. Long Terminal Repeat (LTR) retrotransposons constituted the predominant repeat class (LmacA: 31.44%; LmacB: 30.67%), with LTR-Gypsy (13.06% vs. 12.75%) and LTR-Copia (12.11% vs. 12.22%) as major subtypes (Table S6).

Functional annotations were assigned to >90.84% of LmacA and >90.80% of LmacB genes (Table S7). Annotation of non-coding RNAs identified a total of 13,490 ncRNA genes in LmacA and 8922 in LmacB, including miRNAs, tRNAs, rRNAs, snRNAs, and scaRNAs. The genomic proportions were as follows: rRNAs were most abundant (LmacA: 0.54455%; LmacB: 0.307609%), followed by snRNAs (0.022312% vs. 0.022362%), tRNAs (0.006871% vs. 0.00701%), and miRNAs (0.002364% vs. 0.002336%) (Table S8).

### 3.2. Structural Variations and Chromosomal Rearrangements Between the Two Haplotypes of L. macranthoides

**Figure 3:**
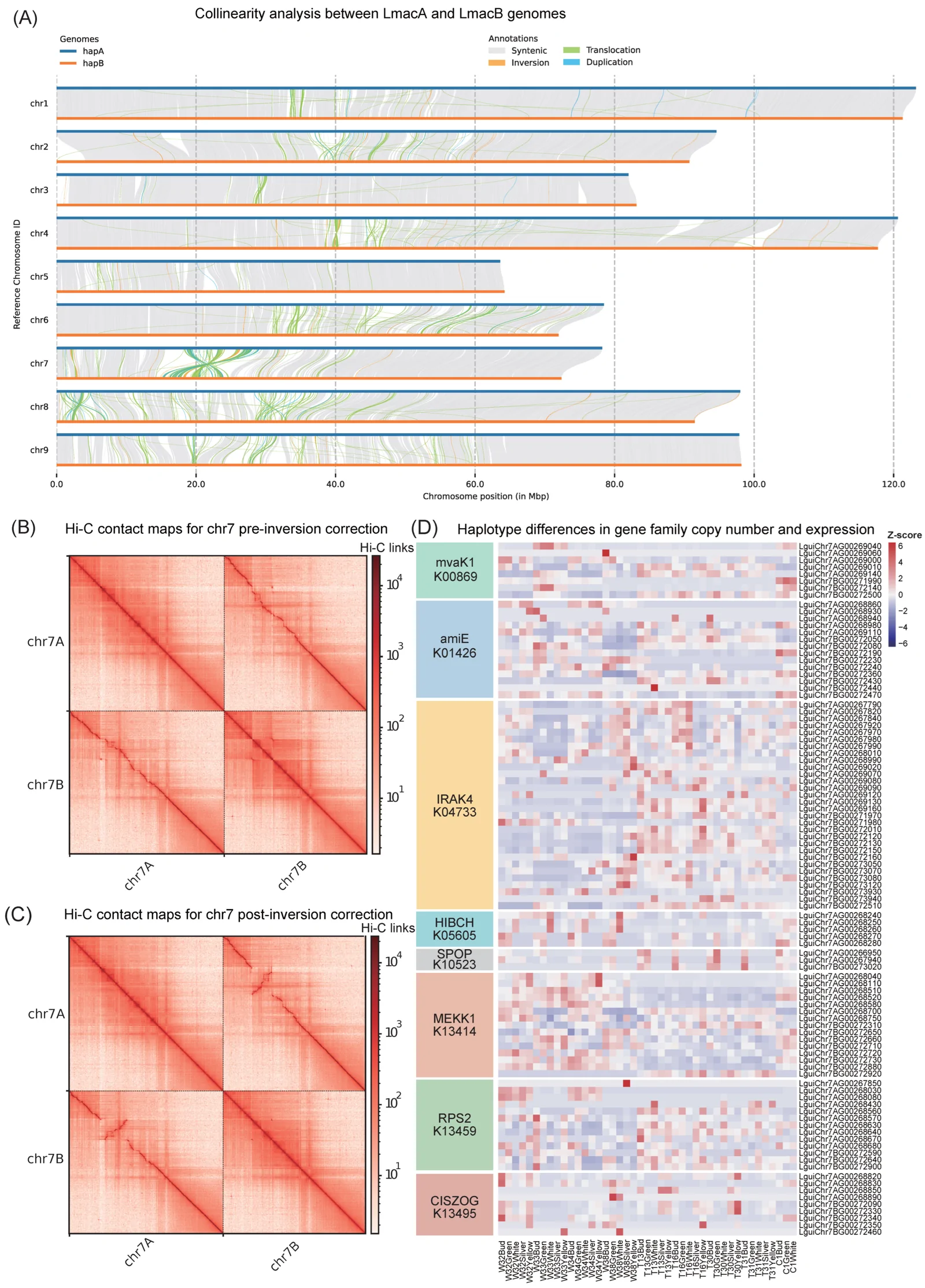
Structural variations in LmacA and LmacB haplomes. (**A**) Collinearity analysis between LmacA and LmacB genomes. The collinear regions between LmacA and LmacB are shown linked by gray lines. Chromosomes from the two haplomes are displayed as horizontal bars, with genomic positions shown in megabases (Mb) along the x-axis and chromosome IDs along the y-axis. (**B**) Hi-C contact maps for chr7 pre-inversion correction. The color bar indicates the number of Hi-C links between chromosome regions on a log10 scale; darker red indicates a higher number of links (i.e., stronger chromatin interactions). (**C**) Hi-C contact maps for chr7 post-inversion correction, using the same scale as in (**B**). (**D**) Haplotype differences in gene family copy number and expression. Rows represent gene families annotated with KEGG ortholog (KO) identifiers; columns represent individual gene copies ordered by chromosome position. Heatmap colors indicate z-score-normalized RNA-seq expression values (red, higher; blue, lower). Data were derived from RNA-seq of flower buds at different developmental stages across cultivars W, T, and C.

Due to the high heterozygosity (1.36%) of the *L. macranthoides* genome, we compared the structure of the two haplotype assemblies (LmacA and LmacB). Using LmacA as the reference, we identified 84,084 structural variations (SVs) (Figure 3A). Most SVs were ≤2 kb in length (Figure S3), with duplications and translocations collectively accounting for 71.4% of events (Table S9). SV structure annotation showed that SVs are mainly concentrated in the intergenic regions (Figure S4). A total of 11,042 genes were defined as SV-affected genes, based on SVs located within exons or introns of annotated genes. KEGG pathway assignment was performed on these 11,042 genes. Of these, 2121 genes had KEGG pathway assignments and were used as the query set. The background comprised the 6825 KEGG-annotated genes from the LmacA representative gene set (36,792 annotated genes in total) (Table S10). The results showed that these genes were mainly assigned to pathways such as Glycosylphosphatidylinositol (GPI)-anchor biosynthesis, Podophyllotoxin biosynthesis, Linoleic acid metabolism in LmacA, and Podophyllotoxin biosynthesis, Sesquiterpenoid and triterpenoid biosynthesis, Glycosaminoglycan degradation, Starch and sucrose metabolism in LmacB (Figure S5, Table S10). The Ka/Ks ratio of both haplotypes was consistent, implying that the two haplotypes experienced similar selection pressure during the domestication history of *L. macranthoides* (Figure S6).

By aligning the two haplotype assemblies with LmacA as the reference, a large inversion (11.32 Mb in LmacA/9.78 Mb in LmacB) was identified on chromosome 7 of both haplotypes, encompassing 2710 and 2080 genes, respectively (Figure 3A). Hi-C contact maps before and after inversion adjustment validated this structural rearrangement (Figure 3B,C). Mevalonate kinase (MVK), *amidase*, interleukin-1 receptor-associated kinase 4 (IRAK4), 3-hydroxyisobutyryl-CoA hydrolase (HIBCH), speckle-type POZ protein (SPOP), mitogen-activated protein kinase kinase kinase 1 (MEKK1), disease resistance protein (RPS2), cis-zeatin O-glucosyltransferase (CISZOG) gene families were annotated in this inversion region. Differential gene copy numbers and expression profiles were observed between haplotypes for these gene families (Figure 3D).

### 3.3. Genome Evolution and Gene Diversification in L. macranthoides

To gain insights into the evolution of the *L. macranthoides* genome, we performed comparative genomic analyses against 13 other plant species, including *L. japonica*, *L. maackii*, *L. caerulea*, *Panax stipuleanatus*, *Apium graveolens*, *Daucus carota*, *Platycodon grandiflorus*, *Chrysanthemum nankingense*, *Lactuca sativa*, *Arctium lappa*, *Ilex polyneura*, *Ilex latifolia*, and *Camellia sinensis*. Using OrthoFinder, we identified a total of 72,741 orthologous gene families (orthogroups) across all species, encompassing 549,696 genes. Among these genes, there were 222,096 genes that belong to 7746 families and are shared across all species. We identified 4356 specific gene families in *L. macranthoides* genome, encompassing 6135 genes (Table S11). KEGG enrichment analysis showed that specific genes of *L. macranthoides* were significantly enriched in pathways such as the biosynthesis of various plant secondary metabolites, cyanoamino acid metabolism, and starch and sucrose metabolism (Figure S7A, Table S12). These enriched pathways are all well known to contribute to plant development and plant response to (a)biotic stresses [64,65,66,67,68,69]. In addition, a large number of these genes were assigned to other pathways like endocytosis and spliceosome, though with lower statistical significance.

**Figure 4:**
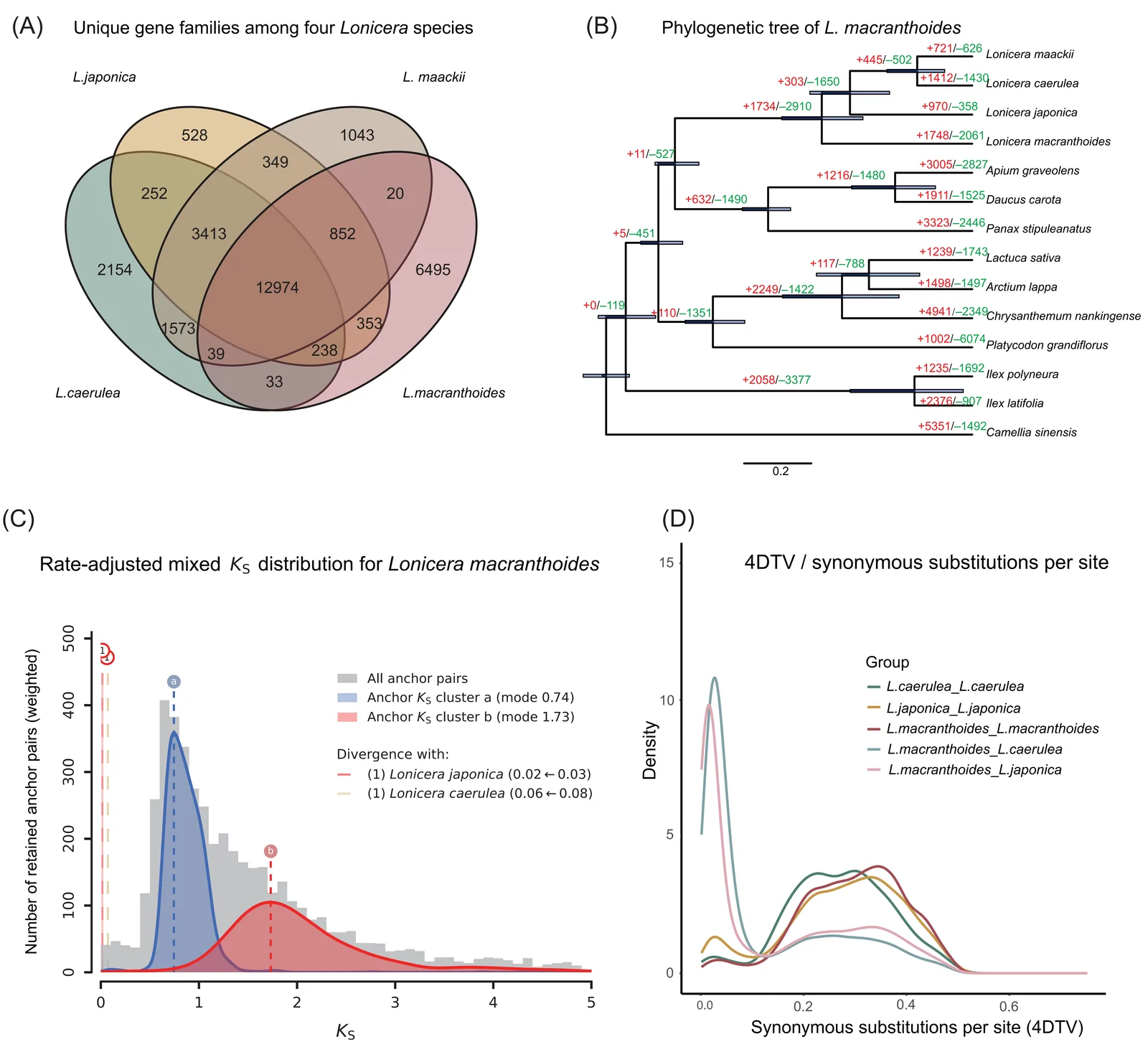
Comparative genomic features of *L. macranthoides*. (**A**) Unique gene families analysis between *L. macranthoides*, *L. japonica*, *L. maackii* and *L. caerulea*. (**B**) Phylogenetic tree of *L. macranthoides*. Inferred phylogenetic tree based on single-copy gene families in 14 plant species. Gene family expansions are indicated in red, and gene family contractions are indicated in green. Blue bars at nodes represent divergence times estimated by Maximum Likelihood (PAML). (**C**) Rate-adjusted mixed Ks distribution for *L. macranthoides* obtained from ksrates. Grey histogram: all anchor pairs; blue curve: anchor Ks cluster a (mode = 0.74); red curve: anchor Ks cluster b (mode = 1.73). Dashed vertical lines indicate divergence with *L. japonica* (Ks = 0.02–0.03) and *L. caerulea* (Ks = 0.06–0.08). X-axis: Ks. Y-axis: Number of retained anchor pairs (weighted). (**D**) Distribution of synonymous substitutions per site (4DTV) for self-comparisons of *L. caerulea*, *L. japonica*, and *L. macranthoides*, and for cross-species comparisons (*L. macranthoides* vs. *L. caerulea*, *L. macranthoides* vs. *L. japonica*). X-axis: Synonymous substitutions per site (4DTV). Y-axis: Density.

Comparative analysis of the gene families across four *Lonicera* species (*L. macranthoides*, *L. japonica*, *L. maackii* and *L. caerulea*) revealed that nearly half (42.8%; 12,974 of 30,316) of the gene families were conserved among all lineages (Figure 4A). *L. macranthoides* shared more orthogroups with *L. japonica* (353) than with *L. maackii* (20) or *L. caerulea*) (33). Enrichment analysis of the common gene families among these four species revealed significant enrichment in pathways related to plant–pathogen interaction and plant response to (a)biotic stress pathways. The most prominent pathways included the MAPK signaling pathway (plant), Phosphatidylinositol signaling system, Plant–pathogen interaction, Stilbenoid, diarylheptanoid and gingerol biosynthesis, Phenylpropanoid biosynthesis, Inositol phosphate metabolism, Glutathione metabolism, and Flavonoid biosynthesis [70,71] (Figure S7B, Table S13). These pathways synthesize secondary metabolites that underpin the medicinal value of *Lonicera* species [72,73,74,75]. *L. macranthoides* possesses the highest number of unique gene families (6495) within the genus. KEGG annotation of lineage-specific genes further highlighted key stress-response pathways: Sesquiterpenoid and triterpenoid biosynthesis, Cyanoamino acid metabolism, Oxidative phosphorylation and Photosynthesis [68,76] (Figure S7C, Table S14).

To clarify the phylogenetic position and evolutionary history of *L. macranthoides*, we identified a total of 1346 single-copy orthogroups across these 14 representative plant species (*L. macranthoides*, *L. japonica*, *L. maackii*, *L. caerulea*, *P. stipuleanatus*, *A. graveolens*, *D. carota*, *P. grandiflorus*, *C. nankingense*, *L. sativa*, *A. lappa*, *I. polyneura*, *I. latifolia*, *and C. sinensis*) for phylogenetic reconstruction. The analysis revealed that *L. macranthoides* diverged earliest among the four *Lonicera* species, followed by *L. japonica*, with all four species forming a monophyletic clade. *L. macranthoides* diverged from *L. japonica* approximately 32.5–56.6 million years ago (Mya), whereas *L. maackii* and *L. caerulea* diverged from their common ancestor around 16.4 Mya, consistent with the previous study [77] (Figure 4B). Following this divergence, 1748 gene families expanded and 2061 contracted in *L. macranthoides* (*p* ≤ 0.05, Figure 4B). KEGG enrichment of these families revealed significant functional associations (Figure S8). Notably, genes in the expanded families were significantly enriched in Sesquiterpenoid and triterpenoid biosynthesis, Flavonoid biosynthesis, Phenylpropanoid biosynthesis, Stilbenoid, diarylheptanoid and gingerol biosynthesis and Ubiquinone and other terpenoid-quinone biosynthesis (Figure S8A, Table S15), whereas genes in the contracted families were mainly enriched in Cutin, suberine and wax biosynthesis, Oxidative phosphorylation, Plant–pathogen interaction and MAPK signaling pathway-plant (Figure S8B, Table S16). Seventeen genes were detected in positive selection analysis and were assigned to pathways such as Fructose and mannose metabolism, Ubiquinone and other terpenoid-quinone biosynthesis, Arginine and proline metabolism, and Plant–pathogen interaction (Figure S8C, Table S17).

WGD events in *L. macranthoides* were investigated using two independent approaches. The ksrates analysis of anchor pairs (Figure 4C) showed a rate-adjusted mixed Ks distribution with two components: a major peak at Ks ≈ 0.74 and a secondary peak at Ks ≈ 1.73 (WGT-γ). The Ks values for divergence from *L. japonica* and *L. caerulea* were much lower (0.02–0.03 and 0.06–0.08, respectively), indicating that these species diverged after the WGD events. In addition, the 4DTV analysis (Figure 4D) revealed a prominent peak at approximately 0.25 for the self-comparison of *L. macranthoides*, which was similar to the patterns observed for *L. japonica* and *L. caerulea*, suggesting a shared recent WGD event within the genus. Together, these results support the occurrence of a recent WGD in *L. macranthoides*, consistent with previous reports in *L. japonica* and *L. caerulea* [17,77].

### 3.4. GWAS for Detecting Variants Associated with Chlorogenic Acid and Dipsacoside B Contents

#### 3.4.1. GWAS and Transcriptome Analysis of CGA Content

**Figure 5:**
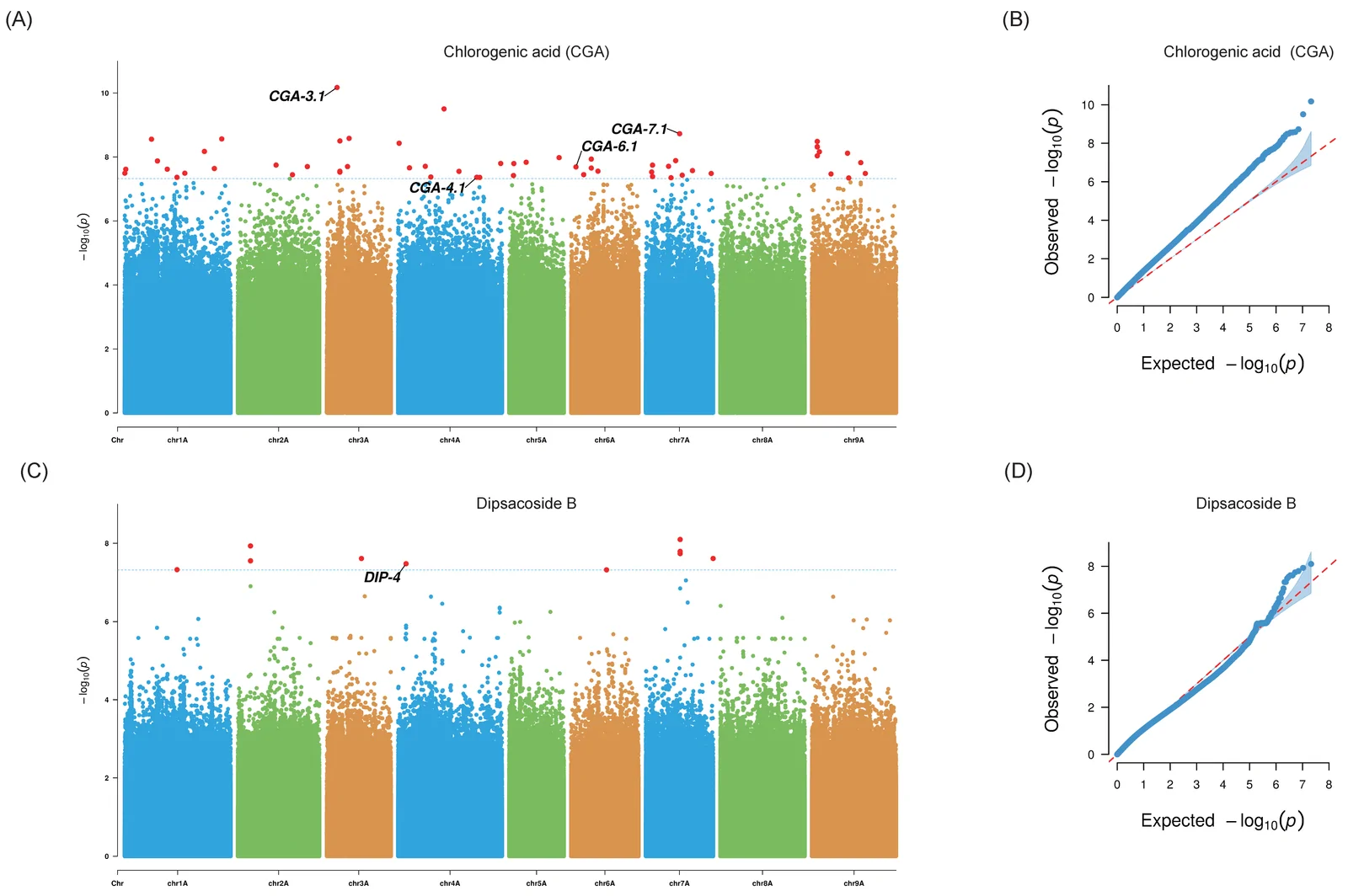
Identification of candidate genes associated with quality traits in *L. macranthoides*. (**A**) GWAS of CGA content. (**B**) Quantile-quantile (Q-Q) plot of the GWAS for CGA content. The dark blue dots indicate SNPs whose observed values deviate from the null expectation (i.e., falling outside the 95% confidence band). The light blue shaded region represents the 95% pointwise confidence band based on the beta distribution under the null hypothesis, indicating the expected normal fluctuation range of *p* values when no true association exists. The red dashed line indicates the theoretical diagonal under the null hypothesis (observed *p* value = expected *p* value). (**C**) GWAS of Dipsacoside B content. (**D**) Q-Q plot of the GWAS for Dipsacoside B content. The dark blue dots indicate SNPs whose observed values deviate from the null expectation (i.e., falling outside the 95% confidence band). The light blue shaded region represents the 95% pointwise confidence band based on the beta distribution under the null hypothesis, indicating the expected normal fluctuation range of *p* values when no true association exists. The red dashed line indicates the theoretical diagonal under the null hypothesis (observed *p* value = expected *p* value).

GWAS identified four signals associated with CGA content in flower buds at the White stage (Figure 5A,B; Table S18). A putative O-methyltransferase gene (*LguiOMT*, *LguiChr3AG00303320*), annotated as a Methyltransferase_3 (3-O-methyltransferase), was identified as the candidate LguiCGA1 gene located downstream of the peak SNP for CGA-3.1. Although caffeoyl-coenzyme A 3-O-methyltransferase (CCoAOMT) participates upstream in the biosynthesis of feruloyl-quinic acids via feruloyl-CoA, it is not directly involved in the CGA pathway; instead, OMT genes indirectly modulate CGA accumulation [71]. The CGA-7.1 region contained a cluster of eleven hydroxycinnamoyl-CoA shikimate hydroxycinnamoyl transferase (HCT) genes (*LguiChr7AG00264070*–*LguiChr7AG00264230*), of which *LguiChr7AG00264160* and *LguiChr7AG00264170* were closest to the peak SNP. It should be noted that this region is located within the Chr7 inversion between LmacA and LmacB; however, the inversion itself was not directly genotyped in the 102 plants, and the associated SNPs therefore represent linked markers rather than the causal structural variant. Putative genes in the CGA-4.1 (*LguiChr4AG00091650*, *LguiCGA5*) and CGA-6.1 (*LguiChr6AG00240760*, *LguiCGA6*) loci encode MYB transcription factors.

Transcriptome analysis across flower developmental stages in three cultivars (W, T, and C) revealed distinct expression patterns for these candidate genes (Tables S19–S23). Correlations between gene expression and metabolite content were computed at the individual-sample level using Pearson and Spearman correlations in two stage windows predefined according to the sampling design: the “Bud-to-White” window shared by all three cultivars (*n* = 9 per cultivar) and the complete developmental series available for W and T (*n* = 15) (Figure S9A, Table S24). *LguiCGA1* expression peaked at the “Bud” stage in C and showed a positive, although non-significant, correlation with CGA accumulation from “Bud” to “White” (Pearson *r* = 0.58, *p* = 0.10, *n* = 9); a marginally significant positive correlation was obtained when the three cultivars were pooled (*r* = 0.40, *p* = 0.039, *n* = 27), whereas no correlation was detected within cultivar W (*r* = 0.20, *p* = 0.61). Its expression remained low across all five stages in T (Figure S9A, Table S24). No sequence variants were detected in the *LguiChr3AG00303320* gene region among the 102 accessions, including its coding sequence and UTRs. We therefore speculate that *LguiCGA1* may regulate CGA content through cis-regulatory elements or epigenetic modifications rather than through coding sequence changes; this hypothesis requires further experimental validation. Among the *HCT* cluster, *LguiChr7AG00264160*, *LguiChr7AG00264170* and *LguiChr7AG00264200* showed the highest expression at the “Bud” stage in W (Table S23), with *LguiChr7AG00264200* displaying the most robust transcript levels. This pattern matches the declining trend of CGA accumulation from the Bud stage onwards in W, although the overall stage effect only approached significance (one-way ANOVA *p* = 0.073; Table S20). At the stage-mean level, *LguiChr7AG00264160* expression correlated significantly with CGA content in W (*r* = 0.92, *p* = 0.029, *n* = 5 stages; Table S22), and at the individual-sample level, *LguiChr7AG00264170* showed the strongest expression–content association in W (*r* = 0.87, *p* = 0.0023, *n* = 9; Table S21). In C, *LguiChr7AG00264170* and *LguiChr7AG00264200* showed higher expression at the “Bud” and “White” stages, corresponding to peaks in CGA accumulation. Five major haplotypes of *LguiChr7AG00264170* were identified, among which HapII exhibited the highest median CGA content (Figure S10, Tables S19 and S20). The two MYB genes at CGA-4.1 and CGA-6.1 exhibited higher expression at the “White”, “Silver”, and “Yellow” stages compared with the “Bud” and “Green” stages across all three cultivars, suggesting that these MYB genes may exert a negative regulatory effect on CGA biosynthesis (Figure S9A, Table S24). Taken together, these results indicate that CGA accumulation is regulated by cultivar-specific and developmental stage-specific expression of OMT, HCT, and MYB genes, with the HCT cluster showing particularly strong haplotype- and cultivar-dependent expression patterns.

#### 3.4.2. GWAS and Transcriptome Analysis of Dipsacoside B Content

For Dipsacoside B, significant associations were detected at multiple loci with similar *p* values; all significant loci from both GWAS analyses—including the DIP-4 lead SNP chr4A_9234533 (*p* = 3.3 × 10^−8^) and, for example, chr1A_60859910 (*p* = 4.7 × 10^−8^)—are listed in Table S18, and the per-model *p* values of all candidate lead SNPs are provided in Table S26 (Figure 5C). The Q-Q plot showed a good fit of the model (Figure 5D). The DIP-4 locus contains eight tandem-duplicated UDP-glucosyltransferase (UGT) genes (*LguiChr4AG00068790*–*LguiChr4AG00068870*), which are key enzymes in the triterpenoid modification process [5].

Dipsacoside B accumulation across cultivars and developmental stages is provided in Table S27. Transcriptome analysis showed that *LguiChr4AG00068810* and *LguiChr4AG00068820* were highly expressed in all tested tissues, while *LguiChr4AG00068840* and *LguiChr4AG00068870* showed positive but non-significant correlations with Dipsacoside B accumulation in W at the individual-sample level (Pearson *r* = 0.41, *p* = 0.12 and *r* = 0.41, *p* = 0.13, respectively, *n* = 15; Table S21); at the stage-mean level, *LguiChr4AG00068840* correlated positively with saponin content in W (*r* = 0.83, *p* = 0.083, *n* = 5; Table S22), whereas no positive correlations were detected in T. In C, *LguiChr4AG00068810* showed a weak positive trend with saponin content (*r* = 0.15, *p* = 0.69; Spearman rho = 0.53, *p* = 0.14; Table S21) (Figure S9B, Table S24). Compared with the two UGT copies previously reported in *L. macranthoides* (EVM0009409, EVM0012591) [17], our identification of eight orthologs demonstrates improved genome assembly completeness. In addition, different UGT genes show dominant expression in different cultivars, which may be due to the selective activation of specific UGT genes by transcription factor combinations or epigenetic modifications. These results indicate that UGT-mediated glycosylation is a key candidate mechanism underlying Dipsacoside B accumulation: the chr4A *UGT* cluster corresponds to the major GWAS signal (DIP-4), and its member genes display cultivar-specific expression patterns that are consistent with the differential saponin accumulation observed among W, T and C (Tables S21, S22 and S27). However, at the individual-sample level, the transcript–metabolite correlations of individual cluster members did not reach statistical significance (Table S21), and the contribution of specific UGT genes to saponin variation therefore requires functional validation.

### 3.5. Developmental Stage and Cultivar-Specific Regulation of Saponin Biosynthesis via Glycosylation Pathways

**Figure 6:**
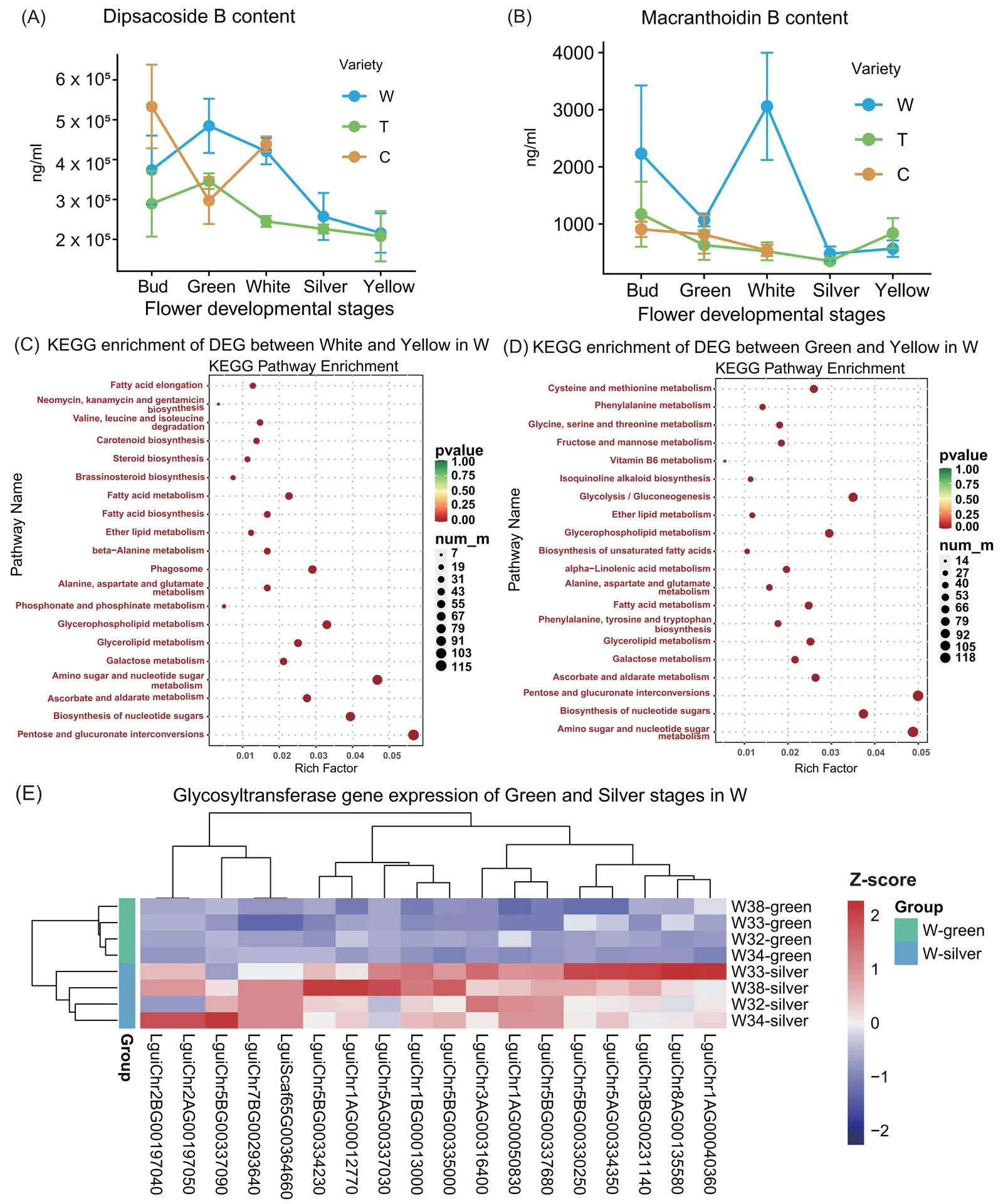
Developmental stage and cultivar-specific regulation of saponin biosynthesis. (**A**) The content of Dipsacoside B in three cultivars at different flower developmental stages. (**B**) The content of Macranthoidin B in three cultivars at different flower developmental stages. Data are presented as mean ± SEM (*n* = 3 biological replicates), and the concentration unit “ng/mL” refers to nanograms of the target metabolite per milliliter of the prepared sample extract. Statistical significance among cultivars at each developmental stage was evaluated using one-way ANOVA, followed by Student’s *t*-test for pairwise comparisons. (**C**) Differentially expressed genes in W-White vs. W-Yellow were significantly enriched in glycosylation-related pathways. The adjusted *p*-values (FDR) for the displayed KEGG pathways range from 1.51 × 10^−12^ to 2.09 × 10^−2^. (**D**): Differentially expressed genes in W-Green vs. W-Yellow were significantly enriched in glycosylation-related pathways. The adjusted *p*-values for the displayed pathways range from 4.43 × 10^−11^ to 2.96 × 10^−3^ (Table S34). (**E**): Heatmap of glycosyltransferase-encoding gene expression in W-Green vs. W-Silver. The color scale represents Z-score-normalized expression values (log2-transformed), ranging from −2 (low expression) to 2 (high expression). Rows represent biological replicates (W32–W38). Columns represent glycosyltransferase genes. As this is a non-model plant lacking officially assigned gene symbols, genes are labeled by their IDs (e.g., *LguiChr2BG00197040*) and a complete annotation list is available in Table S28.

Among the four metabolites quantified by targeted metabolomics, significant cultivar- or developmental-stage-dependent accumulation differences were only observed for Dipsacoside B and Macranthoidin B; by contrast, Macranthoidin A and chlorogenic acid showed no statistically significant variations across cultivars at any given flower-developmental stage. We therefore focused subsequent analysis on Dipsacoside B and Macranthoidin B (Tables S19 and S33). Comparative analysis of Dipsacoside B across flower developmental stages revealed that its content in cultivar W decreased significantly at the “Yellow” stage compared with the “White” (*p* = 0.026) and “Green” stages (*p* = 0.033). At the “Green” stage, W showed the highest Dipsacoside B concentration, followed by T, while C had the lowest (Figure 6A); however, one-way ANOVA revealed no statistically significant differences among the three cultivars at this stage (*p* = 0.105) (Table S33). Cultivar C reached its maximum Dipsacoside B level at the “Bud” stage (Figure 6A). Macranthoidin B content was 2–3 orders of magnitude lower than Dipsacoside B (Figure 6B). In W and T, Macranthoidin B peaked at the “Bud” stage and gradually declined from “White” to “Silver” followed by a slight increase. Notably, W reached its overall peak at the “White” stage (~3050 ng/mL), while T surpassed W at the “Yellow” stage (Figure 6B). No significant differences in Macranthoidin B content were found among the three cultivars at any developmental stage (Table S33).

Taken together, these results revealed a stage-specific reduction in Dipsacoside-B exclusively at the Yellow senescent flower stage in cultivar W, whereas no significant change was observed at the Silver stage. This metabolic alteration was not statistically evident in cultivar T, and cultivar C is a closed-bud type that cannot reach Silver- and Yellow-stage open flowers. Visible fluctuations in Dipsacoside-B across C’s limited developmental window were not supported by statistical tests. Macranthoidin-B showed divergent developmental trajectories across cultivars: T and C displayed a gradual downward trend from the bud stage onward, while W exhibited a distinct peak at the White stage. Collectively, these cultivar-dependent metabolic patterns suggest that saponin metabolic reprogramming is controlled by both cultivar background and flower developmental stage, which motivated our subsequent investigation of glycosylation-related gene expression.

To identify transcriptional drivers of the late-stage saponin decline in cultivar W (Figure 6A,B), we compared W-White vs. W-Yellow and W-Green vs. W-Yellow. A total of 10,316 and 12,912 DEGs were identified respectively, significantly enriched in glycosylation-related pathways (Figure 6C,D, Table S28). The Dipsacoside B reduction at the “Yellow” stage likely results from the downregulation of UDP2 genes (*LguiChr2AG00216330* and *LguiChr2BG00214870*) (Table S29).

To further explore the molecular basis underlying the decrease in Macranthoidin B from W-Green (high Macranthoidin B level) to W-Silver (the minimum point of Macranthoidin B accumulation). W-White was omitted from this analysis due to substantial biological variation (Figure 6B). While Macranthoidin B trended upward from W-Silver to W-Yellow, this change was statistically non-significant and was not addressed by the current contrast. This pairwise comparison revealed a set of glycosyltransferase genes with altered expression between the two stages. Specifically, UGT genes enriched for GO:0016757 (glycosyltransferase activity) were significantly upregulated in W-Silver relative to W-Green (Figure 6E, Table S30). Notably, none of these upregulated glycosyltransferases correspond to the functionally validated LmUGT73P1, the only reported UGT involved in an intermediate glycosylation step of the Macranthoidin B biosynthetic pathway. Therefore, we speculate that the upregulation of these glycosyltransferases may divert shared triterpenoid precursors toward alternative glycosylated metabolites, competing with Macranthoidin B biosynthesis and contributing to its lower abundance at the Silver stage. Following the intra-cultivar analysis of cultivar W described above, we further performed an inter-cultivar comparison T-white_vs._C-white to explore cultivar-specific divergence at the “White” stage. T-white_vs._C-white explored cultivar-specific divergence at the “White” stage (Figure 6A). The UGT gene *LguiChr4AG00091540* was downregulated in C-White, potentially reducing a competing glycosylation branch and promoting Dipsacoside B accumulation in cultivar C (Figure S11, Table S29).

Collectively, our findings indicate that glycosylation mediated by UDP2 and UGTs serves as a key regulatory node governing the biosynthesis and accumulation of Dipsacoside B and Macranthoidin B. The observed cultivar-specific saponin dynamics suggest divergent metabolic strategies among the three cultivars: W may prioritize UDP-glucose allocation to corolla splitting at the expense of saponin synthesis in late stages, and its Silver-stage reduction in Macranthoidin B is potentially driven by the upregulation of competing glycosyltransferases. In contrast, the partially dehiscent cultivar T maintains saponin accumulation in later stages, presumably via a balanced UDP-glucose partitioning [78]. Meanwhile, the non-dehiscent cultivar C exhibits peak saponin levels (Figure 6A,B) at the bud stage, which is supported by the sustained high expression of UDP2/UGT genes and the downregulation of a competing UGT branch (e.g., *LguiChr4AG00091540*) in its transcriptome (Table S29). This coordinated variation between saponin glycosylation and flower development suggests a potential evolutionary trade-off between reproductive development (e.g., corolla dehiscence) and bioactive compound retention. Future multi-omics studies are needed to further validate this resource allocation mechanism in *Lonicera*.

### 3.6. Effects of Plant Hormones on Flower Morphological Development

**Figure 7:**
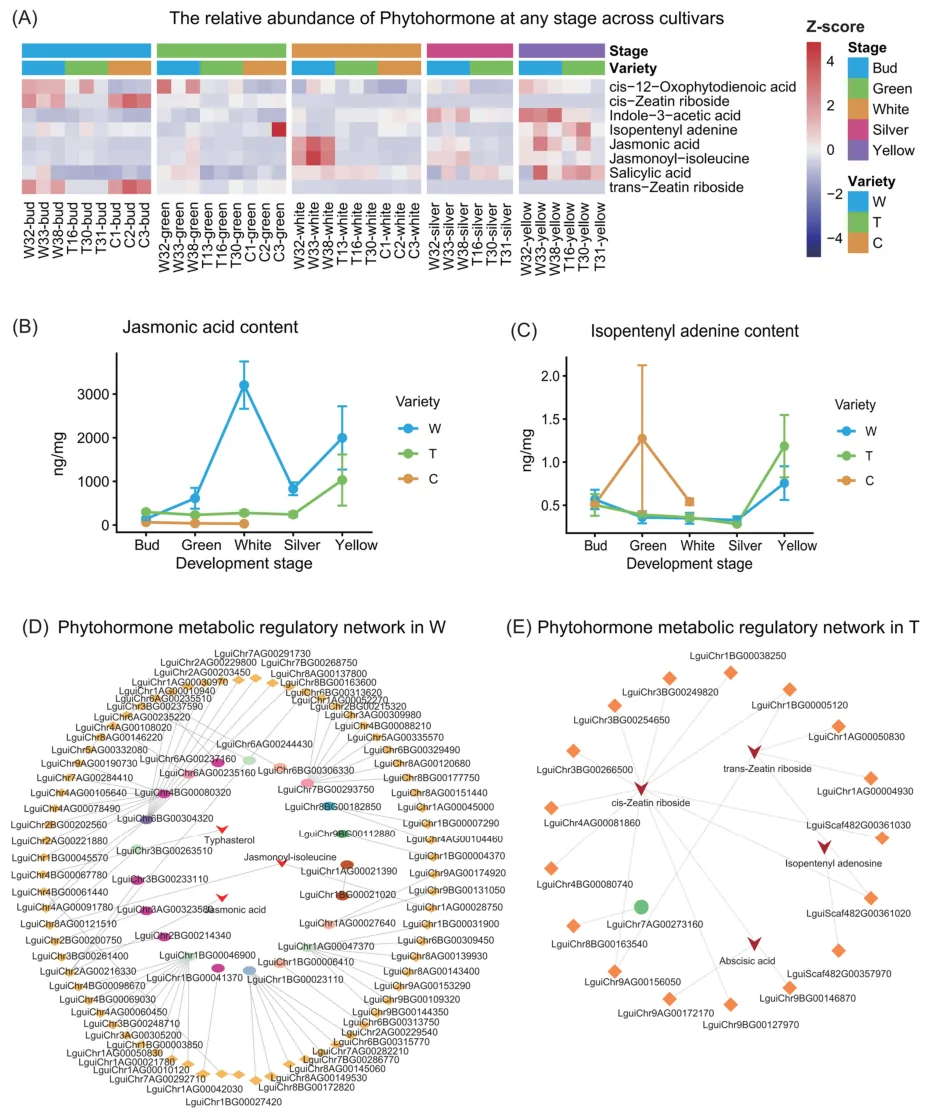
Effects of plant hormones on flower morphological development. (**A**) Heatmap of hormones’ relative content at any stage across cultivars. Heatmap of z-score-normalized hormone content across cultivars and developmental stages (each hormone was z-score standardized, mean = 0, SD = 1; positive/negative values indicate above/below the mean). (**B**) The relative content of Jasmonic acid in three cultivars at different flower developmental stages. (**C**) The relative content of Isopentenyl adenine in three cultivars at different flower developmental stages. (**D**): The TO-GCN temporal co-regulatory network underlying the “White”-to-“Silver” transition in cultivar W. (**E**): The TO-GCN temporal co-regulatory network underlying the “White”-to-“Silver” transition in cultivar T. Structural genes are shown as circles, transcription factors as diamonds, and hormone nodes as triangles in (**D**,**E**).

Phenotypic observations showed distinct floral characteristics among the three cultivars: W exhibits a flowering type, C a closed-bud type, and T an intermediate type with both traits. Previous studies found that differentially expressed genes between cultivars with varying degrees of corolla splitting were mainly involved in phytohormone signal transduction [79,80]. To test this, we performed targeted phytohormone metabolite detection across developmental stages in W, T, and C. The common differentially regulated hormones were cis-zeatin riboside, IAA, isopentenyl adenine (iP), jasmonic acid (JA), salicylic acid, cis-12-oxophytodienoic acid, trans-zeatin riboside, and jasmonoyl-isoleucine (Figure 7A). JA and iP exhibited patterns closely aligned with corolla-splitting phenotypes.

In W, JA levels gradually increased from “Bud” to “White”, declined at “Silver”, and rose again at “Yellow”. By contrast, in T, JA remained low and relatively stable from “Bud” to “Silver” (232–298 ng/g FW) and peaked only at “Yellow” (1030.8 ng/g FW), while C showed the lowest JA content. Overall JA content followed W > T > C (Figure 7B, Table S32). To test how these hormonal dynamics were reflected in the jasmonate-signaling module, we examined the expression of JAZ, MYC2 and JAR1 genes by DESeq2 and correlated their expression with JA (and JA-Ile) content across the developmental stages (Tables S31 and S32). In W, the Green-to-White transition, during which JA reached its maximum, was accompanied by a transcriptional reorganization of this module: the JAZ genes *LguiChr6AG00238270*, *LguiChr6BG00300660* and *LguiChr6AG00231520* were upregulated at White relative to Green, whereas *JAR1_4_6* (*LguiChr8BG00182310*) and *MYC2* (*LguiChr1AG00045820*) were downregulated (Table S32). Consistently, across the five developmental stages, JA-Ile content was positively correlated with *JAZ* expression and JA content was negatively correlated with *JAR1_4_6* (Table S32). In T, the lower and more stable JA levels, peaking only at “Yellow”, were accompanied by the downregulation of the JAZ genes *LguiChr6BG00300660* and *LguiChr6AG00231520* at the Green stage relative to Bud, whereas *JAR1_4_6* and *MYC2* were not differentially expressed, and no stage-mean correlation with JA was significant in T (Table S32). No other jasmonate-pathway genes (e.g., *COI1*, *AOS*, *AOC*, *OPR3*) were differentially expressed in these comparisons, indicating that the association was confined to the *JAR1*–*JAZ*–*MYC2* module. IP content decreased consistently from Bud to Green across all cultivars. Cultivar C showed a relatively large error bar at Green (0.5–2.1 ng/mg), probably owing to biological variability, but the overall downward trend was maintained. C began to recover earlier, at White (~0.5 ng/mg), whereas W and T continued to decline, with W reaching its lowest level at White (~0.3 ng/mg). Thereafter, both W and T rose sharply to maxima at Yellow (~0.75 and ~1.2 ng/mg, respectively) (Figure 7C). To elucidate the molecular basis underlying the dynamic changes in iP in W at the “White” stage, we integrated the transcriptomic data across all cultivars and stages. Correlation analysis between hormone levels and RNA levels revealed that key genes involved in the MVA pathway exhibited transcriptional upregulation in W-White. Specifically, the expression levels of key MVA pathway genes, *HMGCR* (*LguiChr2AG00196720*, *LguiChr2BG00196690*) and *HMGCS* (*LguiChr5AG00342560*), were significantly upregulated in W-White compared to other stages of W and the corresponding stage in C and T (Table S31). Since the MVA pathway supplies isopentenyl diphosphate (IPP) and dimethylallyl diphosphate (DMAPP), the direct precursors for cytokinin (iP) synthesis [77], this transcriptional upregulation likely serves as a preparatory mechanism to accumulate precursors for the subsequent sharp increase in iP at the Yellow stage. Although free iP levels are lowest at this stage, the enhanced RNA levels of MVA genes provide the necessary metabolic foundation for later developmental processes.

To resolve the hierarchical regulatory network governing corolla dehiscence, a co-regulatory network consisting of structural genes, transcription factors and phytohormones was constructed using the TO-GCN temporal co-expression model (Table S35). In W, the network included 179 DEGs, 54 TF genes, and 10 hormones (targeted hormones involved in flower development, including Abscisic acid (ABA) and Typhasterol, it was not restricted to the 8 common differentially regulated hormones across cultivars) (Table S36). During the “White” to “Silver” transition, 72 structural genes involved in JA related regulation showed significant differential expression (48 down-regulated and 24 up-regulated), within the regulatory module governed by 22 TFs, regulated by 22 TFs, predominantly *MYB* (6) and AP2 (4). (Figure 7D; Table S37). These 72 structural genes include a gibberellin-regulated protein (*LguiChr2AG00229800*) and a MAPK family member, MPK7 (*LguiChr1BG00027420*) (Figure 7D; Table S37). In T, 17 structural genes involved in iP and ABA synthesis were highly expressed, regulated by a bHLH TF (*LguiChr7AG00273160*) (Figure 7E; Table S38).

In summary, core transcription factors including MYB, AP2 and bHLH are associated with the regulatory network of corolla dehiscence in *L. macranthoides*, accompanying temporal changes in JA and iP metabolic pathways: the JA pathway appears to be associated with corolla opening and stress responses, and its accumulation level is highly associated with corolla dehiscence phenotype; the cytokinin precursor iP may contribute to the tempo of floral organ development. Together, these processes likely contribute to the phenotypic divergence of corolla dehiscence among different cultivars.

## 4. Discussion

*L. macranthoides* is a major source of the important traditional Chinese medicine Lonicerae Flos, but its genome is highly repetitive and heterozygous, posing challenges for assembly. By combining BGI short reads, PacBio long reads, and Hi-C data, we successfully assembled a high-quality haplotype-resolved genome. The two haplotype assemblies showed significant improvements in continuity and completeness (BUSCO) compared with the previously published *L. macranthoides* genome [17], providing a more comprehensive annotation of gene structures (Table 1) [17].

Phylogenetic analysis clarified the evolutionary relationship among four *Lonicera* species, with divergence times consistent with prior research [77]. Analyses of species-specific gene families across 14 species and focused comparisons within *Lonicera* identified numerous genes associated with plant–pathogen interaction, stress response, and secondary metabolite biosynthesis. These findings demonstrate the robust environmental adaptability and high medicinal potential of *L. macranthoides* at the genomic level [5,74,75].

Comparative analysis of structural variations between LmacA and LmacB revealed that differentially associated genes were primarily involved in podophyllotoxin biosynthesis, linoleic acid metabolism, sesquiterpenoid and triterpenoid biosynthesis, glycosaminoglycan degradation, and starch and sucrose metabolism. Notably, we identified a major inversion event (11.32 Mb in LmacA/9.78 Mb in LmacB) on chromosome 7, containing genes with roles in secondary metabolism (MVK) and stress response (MEKK1) [81]. These gene families exhibited significant differences in copy number and expression between haplotypes, potentially linked to this rearrangement.

CGAs are the primary bioactive components in *L. macranthoides*, with biosynthesis regulated by interconnected factors. Through GWAS, we identified OMTs, HCT, and MYB transcription factors associated with CGA biosynthesis. HCT functions downstream in the CGA pathway, catalyzing the hydroxylation of coumaroyl-CoA to caffeoyl-CoA [6,7,82]. Unlike previous studies, we identified an HCT gene cluster in which many copies remain transcriptionally silent while expressed genes show varietal specificity. This may be due to HCT’s dual role in CGA and lignin synthesis, where substrate competition (e.g., hydroxycinnamoyl-CoA flux) affects CGA content [83,84]. Additionally, differences in cis-elements or epigenetic modifications in the HCT promoter across cultivars may drive spatiotemporal expression. MYB genes identified in GWAS showed a potential negative correlation with CGA content, consistent with the repressor role of some R2R3-MYB members such as *AtMYB4* [85,86]. The correlation analysis showed that the strongest expression-CGA association was observed for the HCT cluster gene *LguiChr7AG00264170* in cultivar W (*r* = 0.87, *p* = 0.0023), whereas the *CCoAOMT* gene *LguiChr3AG00303320* correlated with CGA content in cultivar C and in the pooled three-cultivar dataset but not within W. These GWAS-prioritized candidates merit functional validation in future studies.

Dipsacoside B and Macranthoidin B, are active triterpenoid saponins in *Lonicera*. Their dynamic accumulation is tightly linked to corolla-splitting phenotypes and glycosylation pathways. Dipsacoside B decreased significantly during flowering (“Silver” and “Yellow” stages) in W and T, consistent with prior findings that unsplit corollas accumulate higher bioactive compounds at the bud stage [87]. Macranthoidin B declined from the bud (Green) to the Silver in W, while T surpassed W at late stages. Although C showed lower saponin levels than W early on, its bud-stage accumulation was stable. This likely reflects rapid depletion of UDP-glucose, limiting saponin synthesis during flowering and accelerating petal senescence and corolla splitting [10]. Our findings demonstrate that glycosylation, mediated by UDP2 and UGTs, is the central regulatory node for saponin biosynthesis. The decline in saponin content during flowering correlates with upregulation of UDP2 and UGT genes. Cultivar-specific strategies arise: W prioritizes UDP-glucose for splitting, reducing saponins; T balances allocation to maintain late-stage saponin synthesis; and non-dehiscent C shows reduced expression of competing glycosyltransferase genes (e.g., LguiChr4AG00091540 downregulated in C-White), thereby favoring Dipsacoside B accumulation. This trade-off is mechanistically linked to divergent JA and iP metabolism, representing evolutionary trade-offs between reproductive success and bioactive saponin retention. W exhibits a high-JA strategy driven by JAZ and MYC2, accelerating senescence and splitting but antagonizing saponin glycosylation [88,89]. C maintains low JA and stable glycosylation gene expression, prioritizing saponin storage. T represents an intermediate strategy (Figure 7B,C). Our findings establish that corolla splitting is governed by an antagonistic network where JA promotes opening at the expense of saponin storage, while cytokinin precursors modulate developmental tempo. We note that this glycosylation-centered model, together with the candidate *UGT* cluster on chromosome 4A, is supported by GWAS, transcriptomic and metabolomic associations (Supplementary Tables S18, S21, S22 and S24) but remains association-based; the contribution of individual UGT genes to saponin variation requires functional validation.

We emphasize that the current evidence is largely association-based. Although GWAS, transcriptomic, and metabolomic analyses consistently prioritized candidate genes and pathways, these relationships do not establish causality. The study is constrained by sampling breadth (three cultivar types and defined developmental stages), and by the absence of direct functional perturbation assays (e.g., gene knockout/overexpression or hormone manipulation). Therefore, the proposed regulatory links should be considered testable hypotheses requiring targeted validation at the genetic and biochemical levels. Future work integrating functional genetics, enzyme activity assays, and long-read RNA-seq for full-length isoform resolution may provide deeper insights into post-transcriptional regulation of secondary metabolite biosynthesis and floral development in *L. macranthoides* [90,91,92].

## 5. Conclusions

In conclusion, we generated a haplotype-resolved chromosome-scale genome assembly for *L. macranthoides* and integrated GWAS, transcriptomic, and metabolomic datasets to prioritize candidate genes and pathways associated with chlorogenic acid and triterpenoid saponin accumulation, as well as flower development-related hormonal regulation. These results provide a valuable genomic resource and a set of testable hypotheses for future mechanistic studies and molecular breeding. However, the inferred regulatory relationships remain to be validated by targeted functional experiments.

## Figures and Tables

**Table 1:** Assembly statistics of the genome of *L. macranthoides*.

Assembly	LmacA	LmacB	LM [17]
Total_length (bp)	857,174,655	824,646,850	1,102,470,865
GC_content (%)	34.82%	34.56%	34.51%
Contig N50 (bp)	31,185,602	27,168,000	1,354,012
Scaffold N50 (bp)	97,870,989	91,503,058	82,400,000
Contig Median (bp)	46,147.50	69,489.00	-
Contig Min (bp)	15,629	17,690	-
Contig Max (bp)	98,371,226	76,658,584	18,484,053
Complete BUSCOs © (single-copy/duplicated)	98.90% (92.5%/6.4%)	99.10% (93.1%/6.0%)	94.36% (89.65%/4.71%)

Note: The fourth column (LM [17]) refers to the previously published genome assembly. Single-copy and duplicated BUSCOs are those present in one or multiple copies in the assembly, respectively. “-” indicates data not available.

**Table 2:** Annotation statistics of the genome of *L. macranthoides*.

Annotation	LmacA	LmacB	LM [17]
Total number of genes	36,792	36,433	40,097
Mean gene length (bp)	5830.85 ± 8396.94	5953.76 ± 10,449.06	5213.56
Mean exon length (bp)	328.88 ± 503.96	328.25 ± 504.01	265
Mean intron length (bp)	1054.87 ± 2681.67	1080.01 ± 3105.21	716.9
Total number of exons	182,662	181,510	-
Total number of introns	145,870	145,077	-
Complete BUSCOs © (single-copy/duplicated)	99.00% (93.9%/5.1%)	99.00% (94.1%/4.9%)	-

Note: The fourth column (LM [17]) refers to the previously published genome assembly. Single-copy and duplicated BUSCOs as those change to are present in one or multiple copies in the assembly, respectively. Gene length is defined as the total genomic span from the start codon to the stop codon, including introns. mRNA length is defined as the total length of exons. Values for lengths are presented as mean ± standard deviation (SD). “-” indicates data not available.

## Data Availability

The data and plant materials presented in this study are available upon request from the corresponding author. This Whole Genome Shotgun project has been deposited at GenBank under the BioProject accession number PRJNA1177167 (Haplotype A) and PRJNA1177166 (Haplotype B) with BioSample accession number SAMN44371390. The data are publicly accessible at https://www.ncbi.nlm.nih.gov/bioproject/PRJNA1177167, https://www.ncbi.nlm.nih.gov/bioproject/PRJNA1177166, and https://www.ncbi.nlm.nih.gov/biosample/SAMN44371390 (accessed on 27 January 2026).

## Supplementary Materials

The following supporting information can be downloaded at: https://www.mdpi.com/article/10.3390/biology15212023/s1, Figure S1: 19-mer frequency distribution of *L. macranthoides* genome sequencing; Figure S2: Gene prediction comparisons and intron characterization in *L. macranthoides*; Figure S3: The distribution of SVs length. Figure S4: The location of the SV in the genes. Figure S5: KEGG enrichment of genes affected by SVs; Figure S6: The Ka/Ks ratio of LmacA and LmacB; Figure S7: KEGG enrichment of specific gene of *L. macranthoides*; Figure S8: KEGG enrichment of evolutionary gene set of *L. macranthoides*; Figure S9: Expression of genes associated with GWAS signals for quality traits; Figure S10: Haplotype analysis of *LguiChr7AG00264170*; Figure S11: Combined analysis of transcriptomics and metabolomics in T-White vs. C-White and heat map of DEG; Figure S12: Combined analysis of transcriptomics and metabolomics in W-Green vs. W-Silver; Table S1: Genome sources of 13 additional species in Evolutionary analyses; Table S2: Primers for-RT-Qpcr; Table S3: Bioinformatics tool information used by the study; Table S4: Sequencing data statistics of *L. macranthoides*; Table S5: Chromosome data statistics of *L. macranthoides*; Table S6: Summary of Repeat contents in *L. macranthoides* genome; Table S7: Functional annotation of *L. macranthoides* genome; Table S8: Summary of noncoding RNA in *L. macranthoides* genome; Table S9: Different types of SVs; Table S10: KEGG enrichment of genes affected by SVs; Table S11: The specific genes in *L. macranthoides* genome; Table S12: KEGG enrichment of specific genes of *L. macranthoides* among 14 species; Table S13: KEGG enrichment of the common gene families among these 4 *Lonicera* species; Table S14: KEGG enrichment of specific gene of *L. macranthoides* among 4 *Lonicera* species; Table S15: KEGG enrichment of expanded genes of *L. macranthoides*; Table S16: KEGG enrichment of contracted genes of *L. macranthoides*; Table S17: KEGG enrichment of genes under positive selection of *L. macranthoides*; Table S18: Summary of GWAS signals for CGA and Dipsacoside B content; Table S19: Raw metabolite concentration data; Table S20: Chlorogenic acid (CGA) content in flower buds of three *L. macranthoides* cultivars (W, T and C) across flower developmental stages; Table S21: Correlations between candidate gene expression and metabolite (CGA or Dipsacoside B) content at the individual-sample level, computed with Pearson and Spearman correlations in two stage windows predefined a priori according to the sampling design: the ‘Bud-to-White’ window shared by all three cultivars (*n* = 9 per cultivar) and the complete developmental series available for W and T (*n* = 15); Table S22: Correlations between candidate gene expression and metabolite content at the stage-mean level (each point is the mean of three biological replicates per stage; *n* = number of developmental stages); Table S23: Expression profiles (TPM; mean ± SD and individual replicates) of GWAS candidate genes across cultivars and flower developmental stages; Table S24: Expression of genes associated with chlorogenic acid and dipsacoside B contents identified by GWAS; Table S25: Haplotype analysis of *LguiChr7AG00264170*; Table S26: *p* values of Dipsacoside B candidate lead SNPs across the six association models (EMMAX, GEMMA-LM, GEMMA-LMM, rMVP-GLM, rMVP-MLM and rMVP-FarmCPU). NA: not reported in the corresponding model output; Table S27: Dipsacoside B content in flower buds of three *L. macranthoides* cultivars (W, T and C) across flower developmental stages. Values are mean ± SD of three biological replicates; individual values are shown; Table S28: Differentially expressed genes per comparison (DESeq2, Padj < 0.05 & |log2FC| > 1); Table S29: Regulation of Saponin Biosynthesis; Table S30: Glycosyltransferase gene expression of Green and Silver stages in W; Table S31: Regulation of plant hormones; Table S32: JA/JA-Ile content across developmental stages (raw quantitative measurement) and Pearson correlation with jasmonate-signaling gene expression; Table S33: Summary of statistical comparisons for metabolite content analyses; Table S34: KEGG pathway enrichment analysis of differentially expressed genes in W-Yellow vs. W-White and W-Yellow vs. W-Green; Table S35: Hormone synthesis/signaling- and flowering-related genes shared between adjacent developmental-stage; Table S36: Core nodes of the TO-GCN temporal co-regulatory network underlying corolla dehiscence in cultivar W; Table S37: Node and edge lists of the TO-GCN temporal co-regulatory network underlying the “White”-to-“Silver” transition in cultivar W; Table S38: Genes in the TO-GCN co-regulatory network underlying the iP/ABA-related module in cultivar T during the “White”-to-“Silver” transition.

## Abbreviations

The following abbreviations are used in this manuscript:

CGAs	chlorogenic acids
HQT	hydroxycinnamoyl-CoA quinate transferase
HCT	hydroxycinnamoyl-CoA shikimate and quinate hydroxycinnamoyl transferase
GWAS	Genome-wide association studies
