# Supplementary material for: Haplotype-Resolved Chromosome-Scale Genome Assembly of Lonicera macranthoides Hand.-Mazz. Reveals Genetic Determinants of Bioactive Compound Biosynthesis and Phytohormone-Mediated Metabolic Regulation
Source: Biology (Basel). 2026 Nov 6;15(21):2023. doi: 10.3390/biology15212023 (PMC13762432; doi:10.3390/biology15212023)
Supplement: Supplementary file 1 [file biology-15-02023-s001.zip › Supplementary Figures-final.pdf]

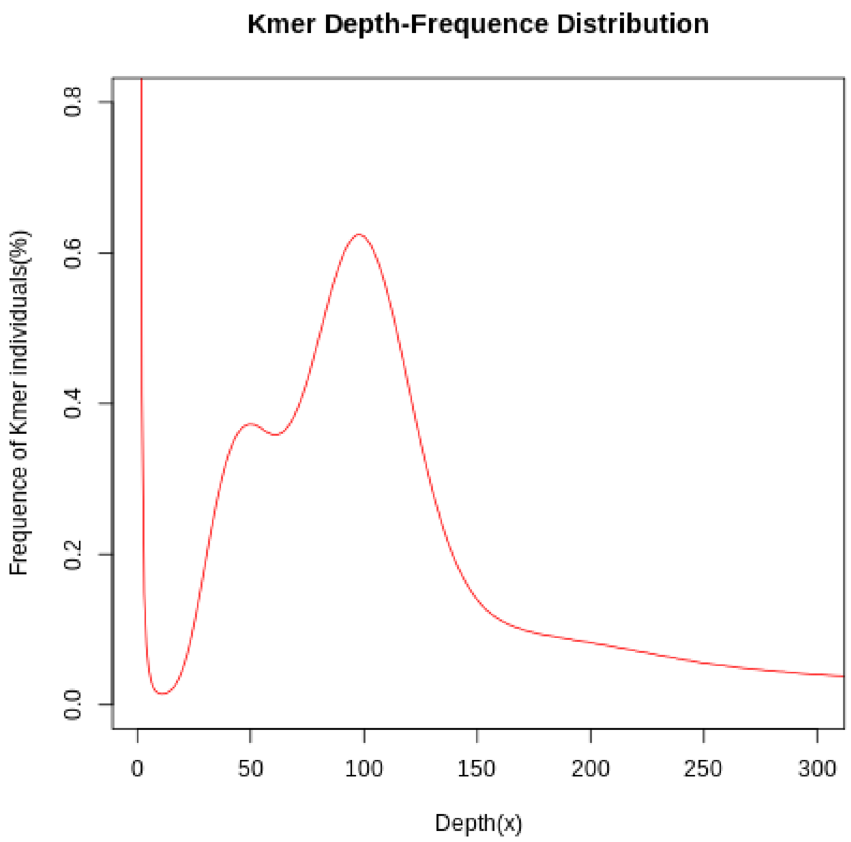

Figure S1 19-mer frequency distribution of *L. macranthoides* genome sequencing. The x-axis represents k-mer depth, and the y-axis represents the frequency of each k-mer depth. The main peak corresponds to the homozygous genomic regions, and the small peaks at approximately half or double the main peak depth correspond to heterozygous regions and repetitive sequences, respectively. Based on this distribution, GCE (version 1.1.0) estimated a genome size of 804.88 Mb, a heterozygosity of 1.36%, and a preliminary repetitive sequence content of 49.67%.

A

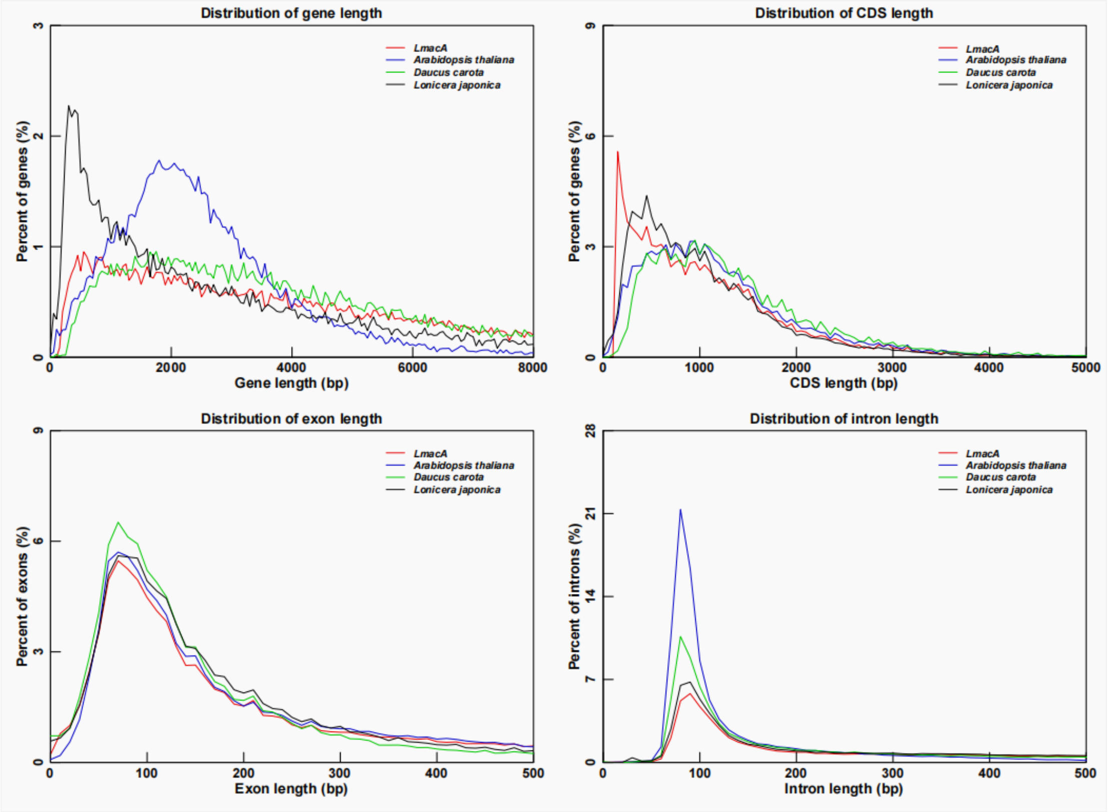

Distribution of gene length
Percent of genes (%)
Gene length (bp)
LmacA
Arabidopsis thaliana
Daucus carota
Lonicera japonica
Distribution of CDS length
Percent of genes (%)
CDS length (bp)
Distribution of exon length
Percent of exons (%)
Exon length (bp)
Distribution of intron length
Percent of introns (%)
Intron length (bp)

B

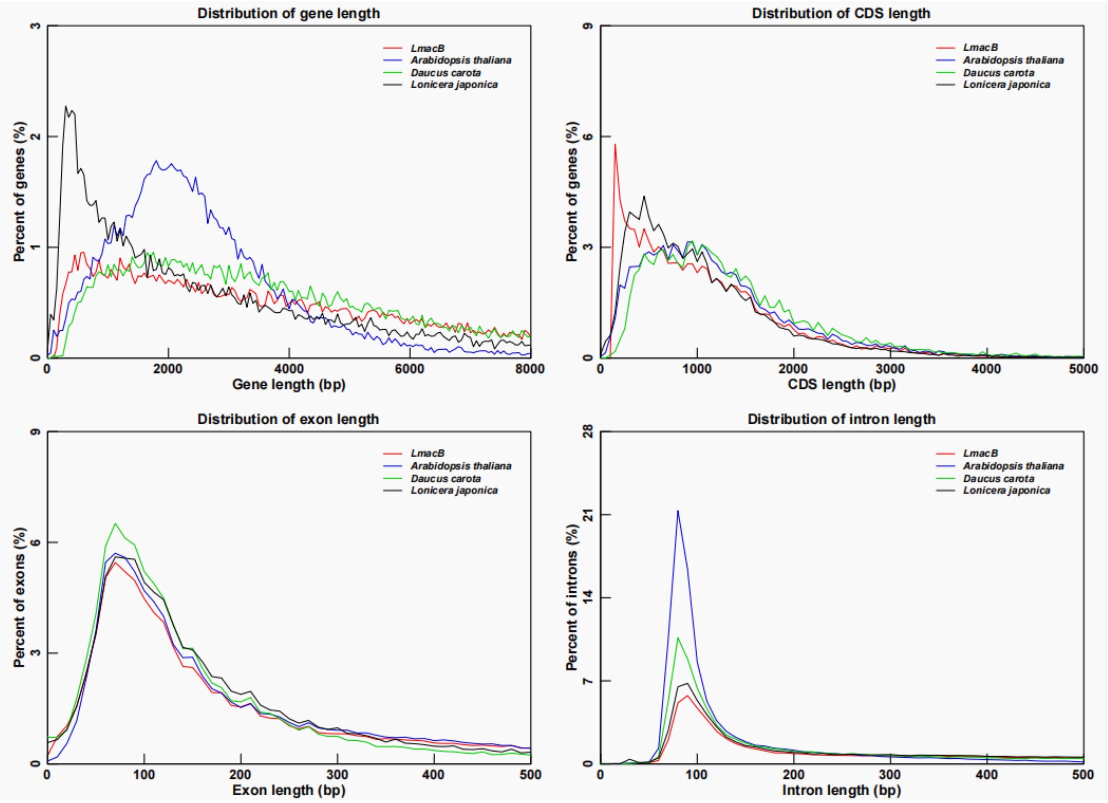

Distribution of gene length
Percent of genes (%)
Gene length (bp)
LmacB
Arabidopsis thaliana
Daucus carota
Lonicera japonica
Distribution of CDS length
Percent of genes (%)
CDS length (bp)
Distribution of exon length
Percent of exons (%)
Exon length (bp)
Distribution of intron length
Percent of introns (%)
Intron length (bp)

C

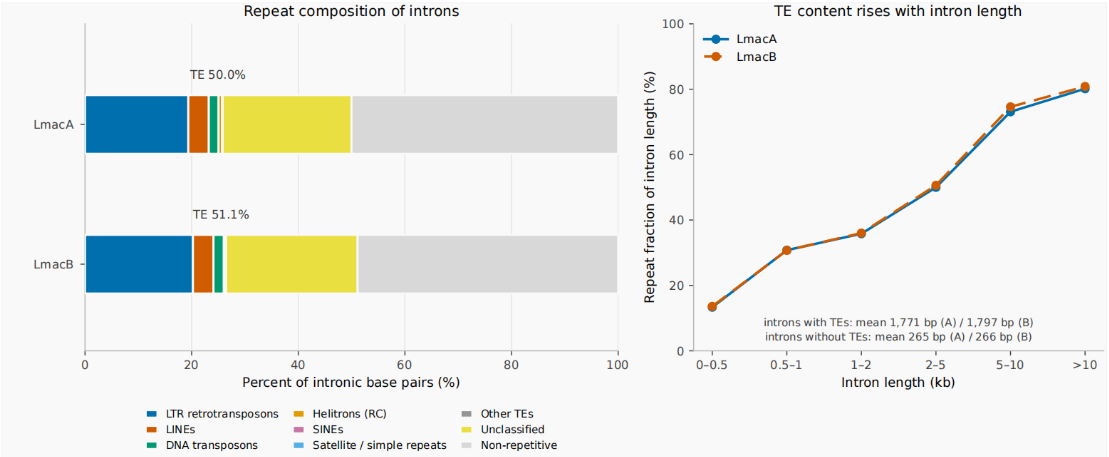

D

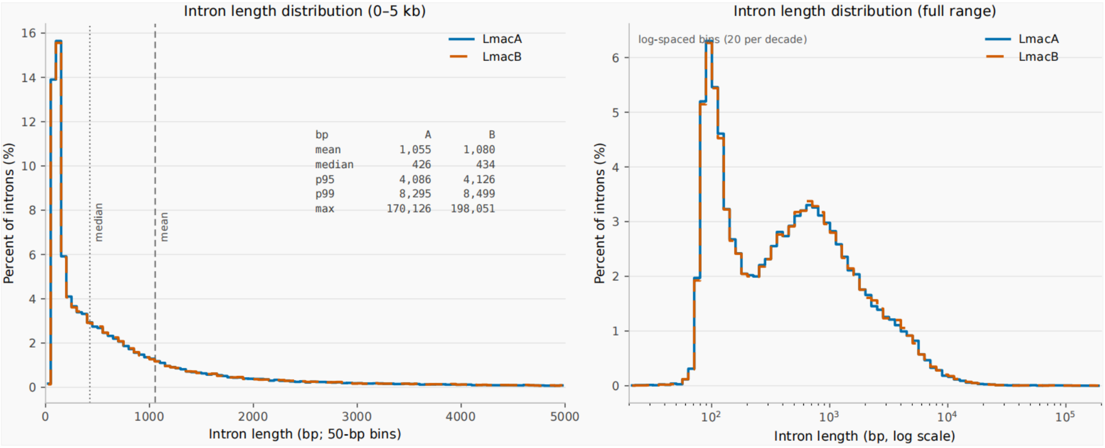

Figure S2 Gene prediction comparisons and intron characterization in *L. macranthoides*.

(A) Distributions of gene length, CDS length, exon length, and intron length for LmacA and the other related species (*Arabidopsis thaliana, L. japonica,* and *Daucus carota*).

(B) The corresponding distributions of gene length, CDS length, exon length, and intron length for LmacB and the other related species. Note that due to the high similarity between the two haplotypes of *L. macranthoides*, the distribution patterns in (A) and (B) are highly comparable.

(C) Intronic repeat composition and its relationship with intron length. Left: Repeat composition of introns, showing that transposable elements (TEs) account for approximately 50% (LmacA) and 51.1% (LmacB) of the intronic base pairs. Right: The repeat fraction increases proportionally with intron length. The mean length of introns

containing TEs (1,771 bp for LmacA and 1,797 bp for LmacB) is substantially longer than that of TE-free introns (265 bp for LmacA and 266 bp for LmacB).

(D) Intron length distribution. Left: Distribution of intron lengths ranging from 0 to 5 kb. The dashed lines indicate the median (426 bp for LmacA and 434 bp for LmacB) and mean (1,055 bp for LmacA and 1,080 bp for LmacB) lengths. Right: Distribution of intron lengths across the full range (log-spaced). The long-tail effect reveals the presence of extremely long introns, with maximum lengths exceeding 170 kb and 198 kb for LmacA and LmacB, respectively.

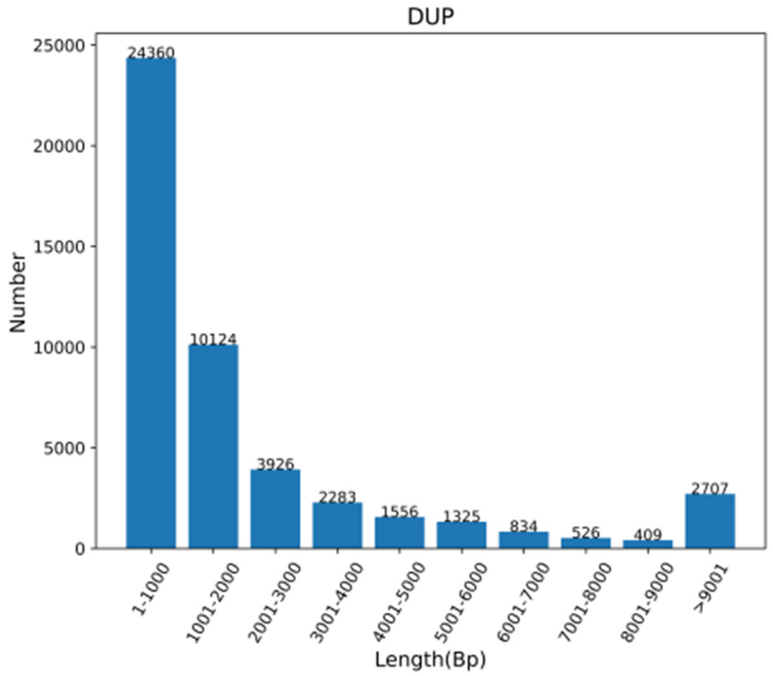

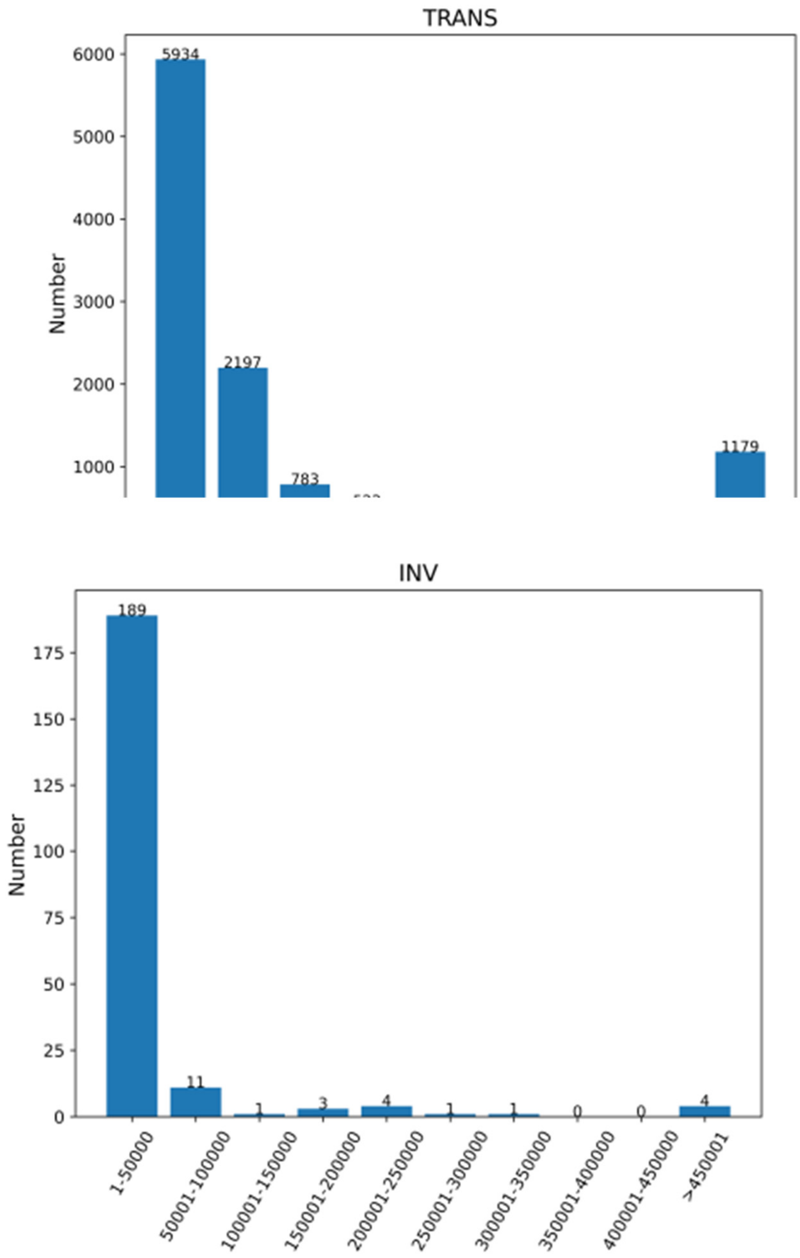

Figure S3 The distribution of SVs length.

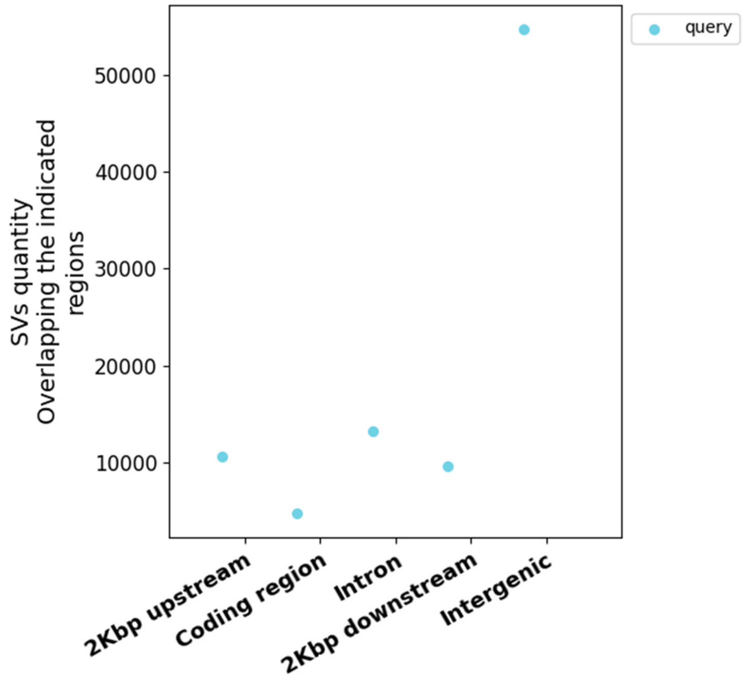

Figure S4 The location of the SV in the genes.

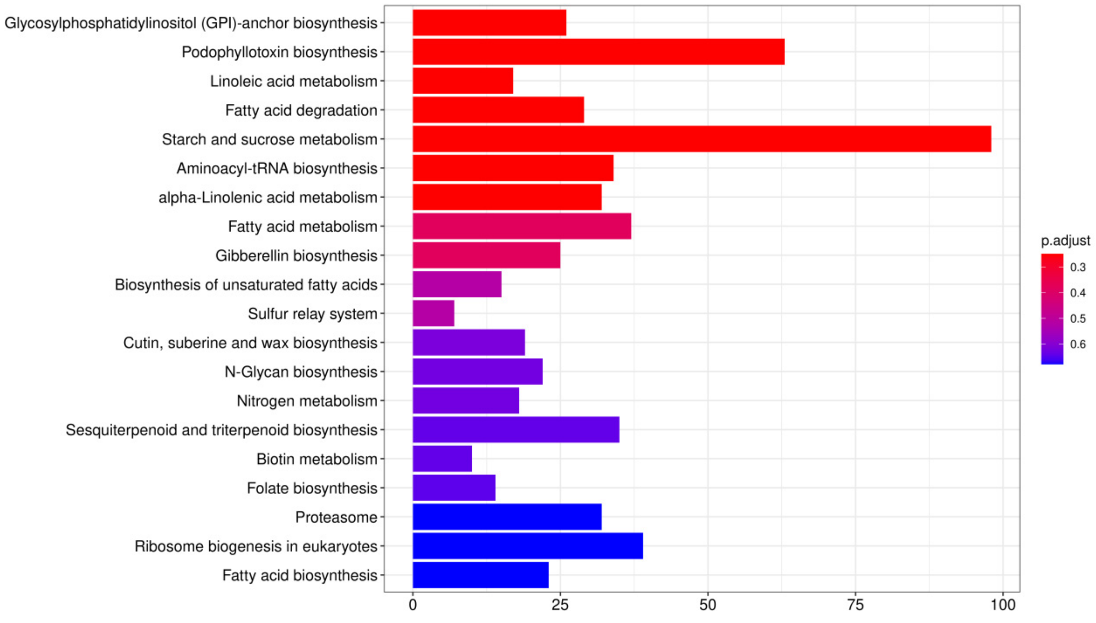

Figure S5 KEGG enrichment of genes affected by SVs.

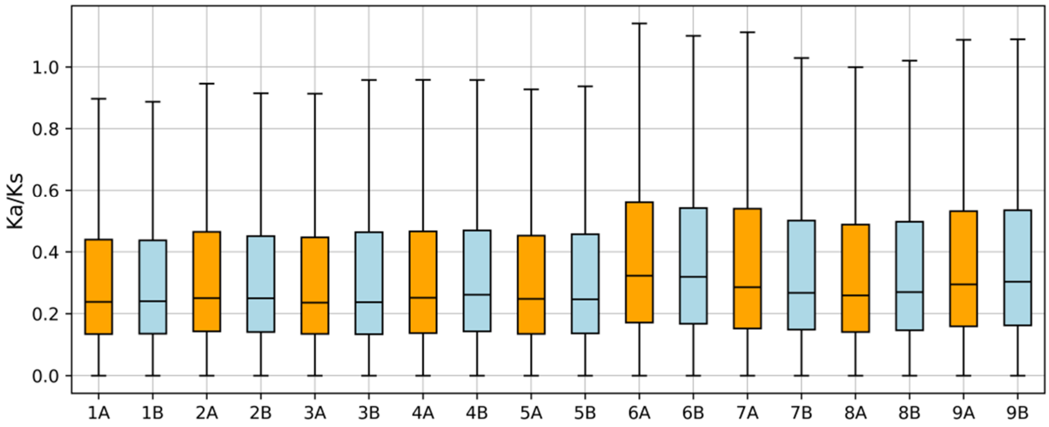

Figure S6 The Ka/Ks ratio of LmacA and LmacB.

A

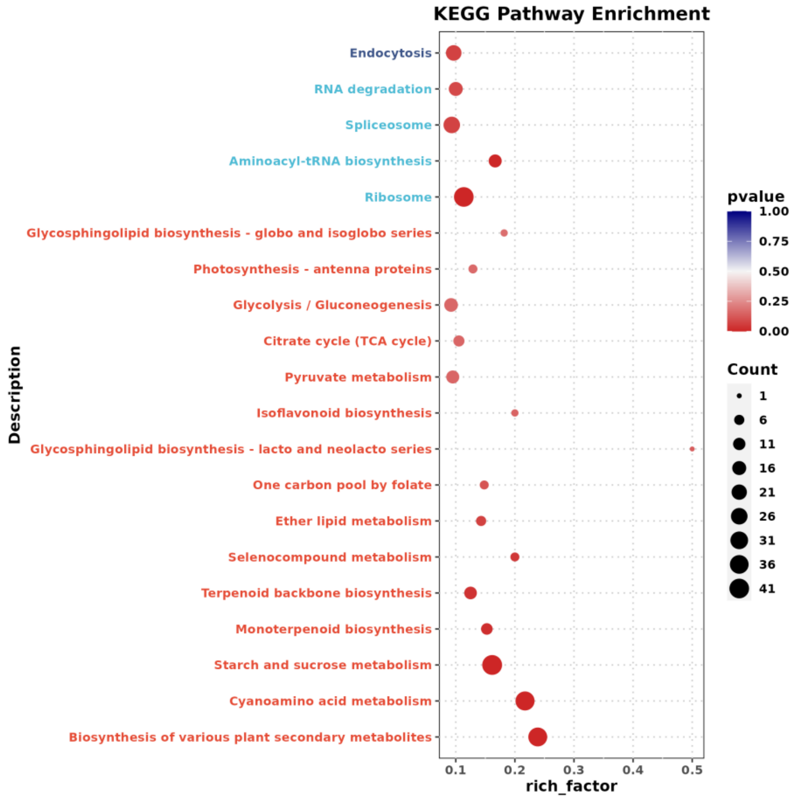

B

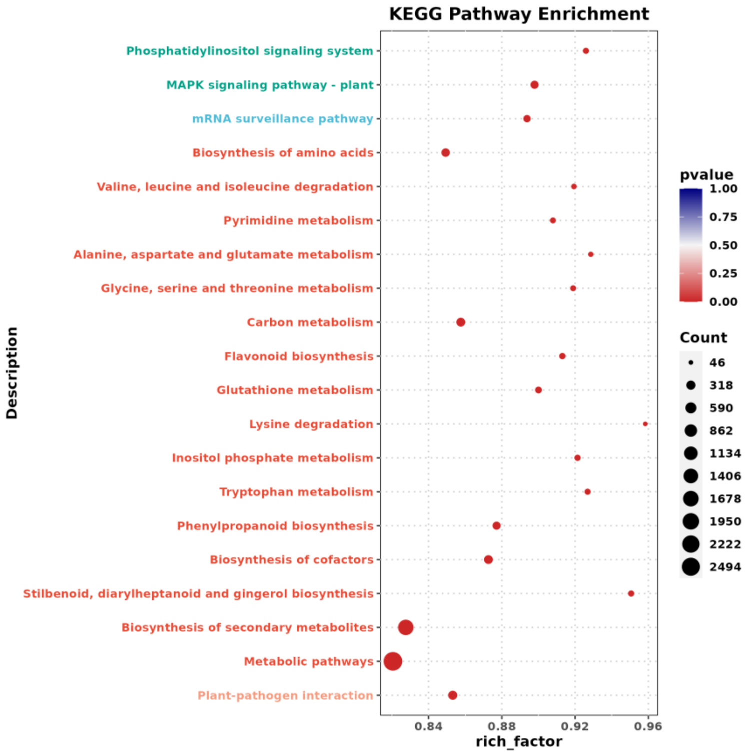
KEGG Pathway Enrichment
Phosphatidylinositol signaling system
MAPK signaling pathway - plant
mRNA surveillance pathway
Biosynthesis of amino acids
Valine, leucine and isoleucine degradation
Pyrimidine metabolism
Alanine, aspartate and glutamate metabolism
Glycine, serine and threonine metabolism
Carbon metabolism
Flavonoid biosynthesis
Glutathione metabolism
Lysine degradation
Inositol phosphate metabolism
Tryptophan metabolism
Phenylpropanoid biosynthesis
Biosynthesis of cofactors
Stilbenoid, diarylheptanoid and gingerol biosynthesis
Biosynthesis of secondary metabolites
Metabolic pathways
Plant-pathogen interaction
Description
0.84
0.88
0.92
0.96
rich_factor
pvalue
1.00
0.75
0.50
0.25
0.00
Count
46
318
590
862
1134
1406
1678
1950
2222
2494

C

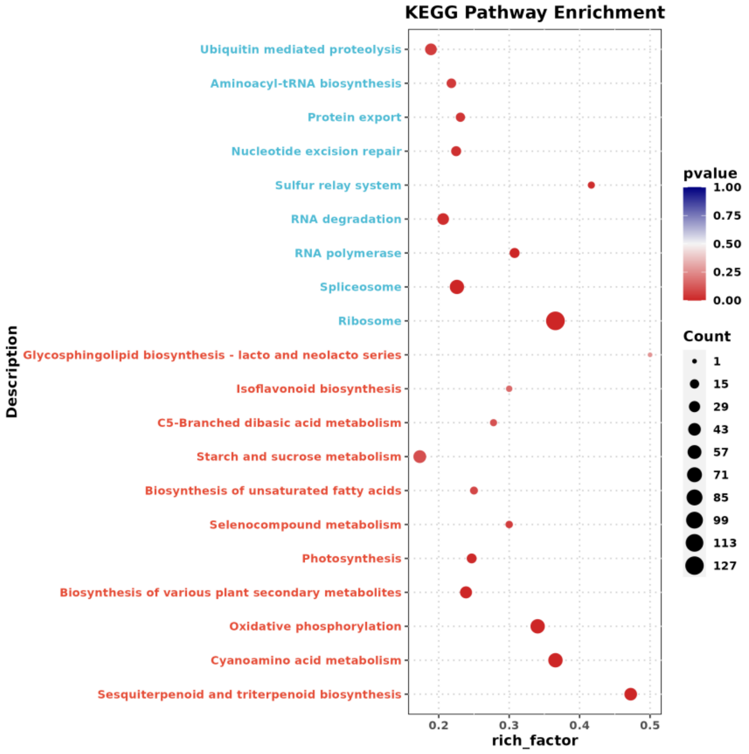
KEGG Pathway Enrichment
Ubiquitin mediated proteolysis
Aminoacyl-tRNA biosynthesis
Protein export
Nucleotide excision repair
Sulfur relay system
RNA degradation
RNA polymerase
Spliceosome
Ribosome
Glycosphingolipid biosynthesis - lacto and neolacto series
Isoflavonoid biosynthesis
C5-Branched dibasic acid metabolism
Starch and sucrose metabolism
Biosynthesis of unsaturated fatty acids
Selenocompound metabolism
Photosynthesis
Biosynthesis of various plant secondary metabolites
Oxidative phosphorylation
Cyanoamino acid metabolism
Sesquiterpenoid and triterpenoid biosynthesis
Description
0.2
0.3
0.4
0.5
rich_factor
pvalue
1.00
0.75
0.50
0.25
0.00
Count
1
15
29
43
57
71
85
99
113
127

Figure S7 KEGG enrichment of specific gene of *L. macranthoides*. A: KEGG enrichment of specific gene of *L. macranthoides* among 14 species. B: KEGG enrichment of the common gene families among these 4 *Lonicera* species. C: KEGG enrichment of specific gene of *L. macranthoides* among 4 *Lonicera* species. The dot plot shows the top enriched KEGG pathways. The X-axis indicates the Rich factor (the ratio of specific genes to background genes in each pathway), the size of the dots represents the number of specific genes (Count), and the color gradient indicates the P value (red indicates higher significance with lower P values, while blue indicates lower significance).

A

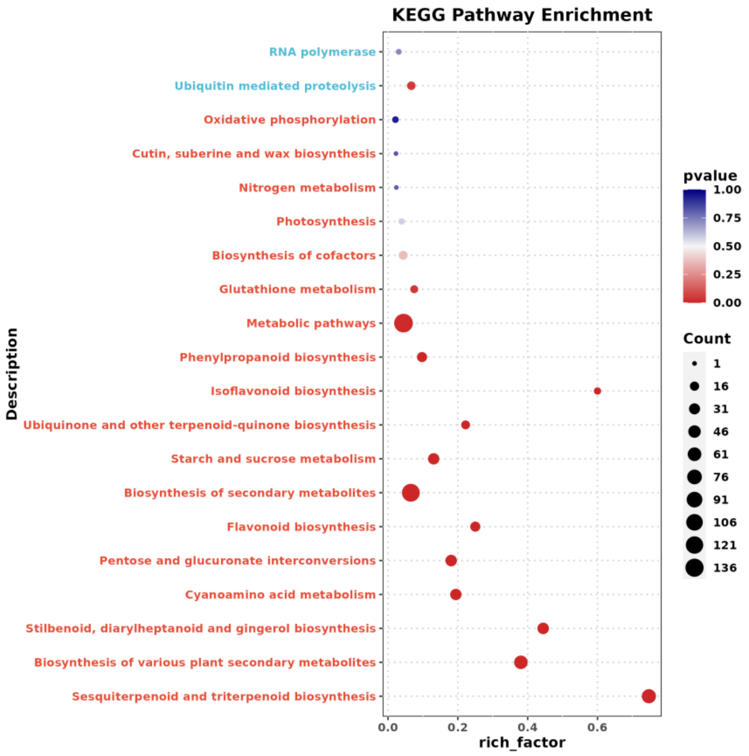
KEGG Pathway Enrichment
RNA polymerase
Ubiquitin mediated proteolysis
Oxidative phosphorylation
Cutin, suberine and wax biosynthesis
Nitrogen metabolism
Photosynthesis
Biosynthesis of cofactors
Glutathione metabolism
Metabolic pathways
Phenylpropanoid biosynthesis
Isoflavonoid biosynthesis
Ubiquinone and other terpenoid-quinone biosynthesis
Starch and sucrose metabolism
Biosynthesis of secondary metabolites
Flavonoid biosynthesis
Pentose and glucuronate interconversions
Cyanoamino acid metabolism
Stilbenoid, diarylheptanoid and gingerol biosynthesis
Biosynthesis of various plant secondary metabolites
Sesquiterpenoid and triterpenoid biosynthesis
Description
0.0
0.2
0.4
0.6
rich_factor
pvalue
1.00
0.75
0.50
0.25
0.00
Count
1
16
31
46
61
76
91
106
121
136

B

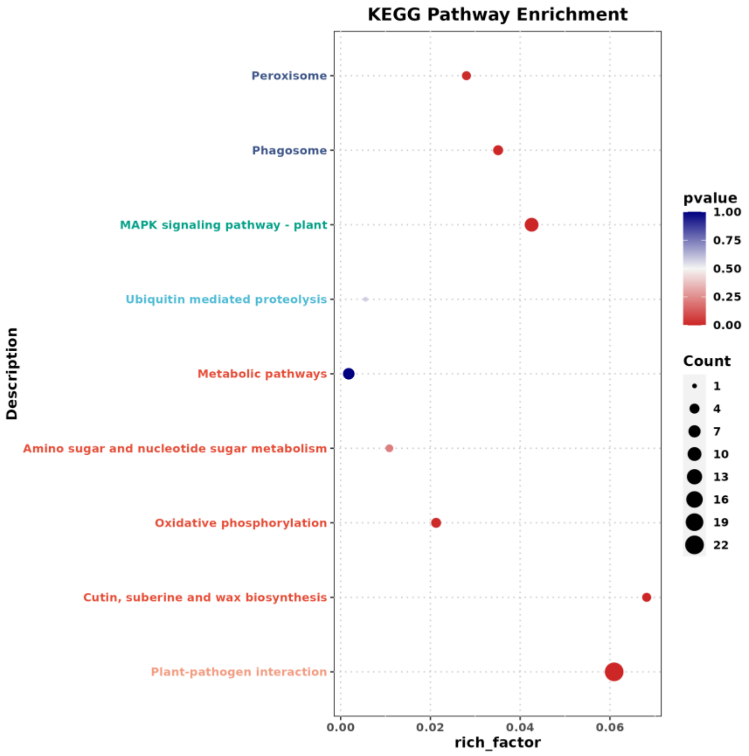
KEGG Pathway Enrichment
Peroxisome
Phagosome
MAPK signaling pathway - plant
Ubiquitin mediated proteolysis
Metabolic pathways
Amino sugar and nucleotide sugar metabolism
Oxidative phosphorylation
Cutin, suberine and wax biosynthesis
Plant-pathogen interaction
Description
0.00
0.02
0.04
0.06
rich_factor
pvalue
1.00
0.75
0.50
0.25
0.00
Count
1
4
7
10
13
16
19
22

C

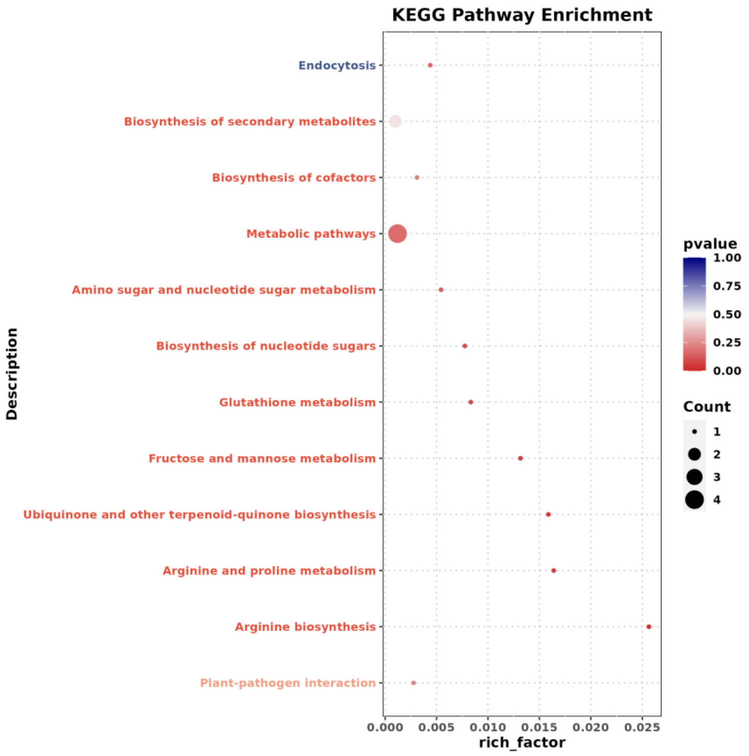

Figure S8 KEGG enrichment of evolutionary gene set of *L. macranthoides.* **A**: KEGG enrichment of expanded genes of *L. macranthoides.* **B**: KEGG enrichment of contracted genes of *L. macranthoides.* **C**: KEGG enrichment of genes under positive selection of *L. macranthoides.* The dot plot shows the top enriched KEGG pathways. The X-axis indicates the Rich factor (the ratio of specific genes to background genes in each pathway), the size of the dots represents the number of specific genes (Count), and the color gradient indicates the P value (red indicates higher significance with lower P values, while blue indicates lower significance).

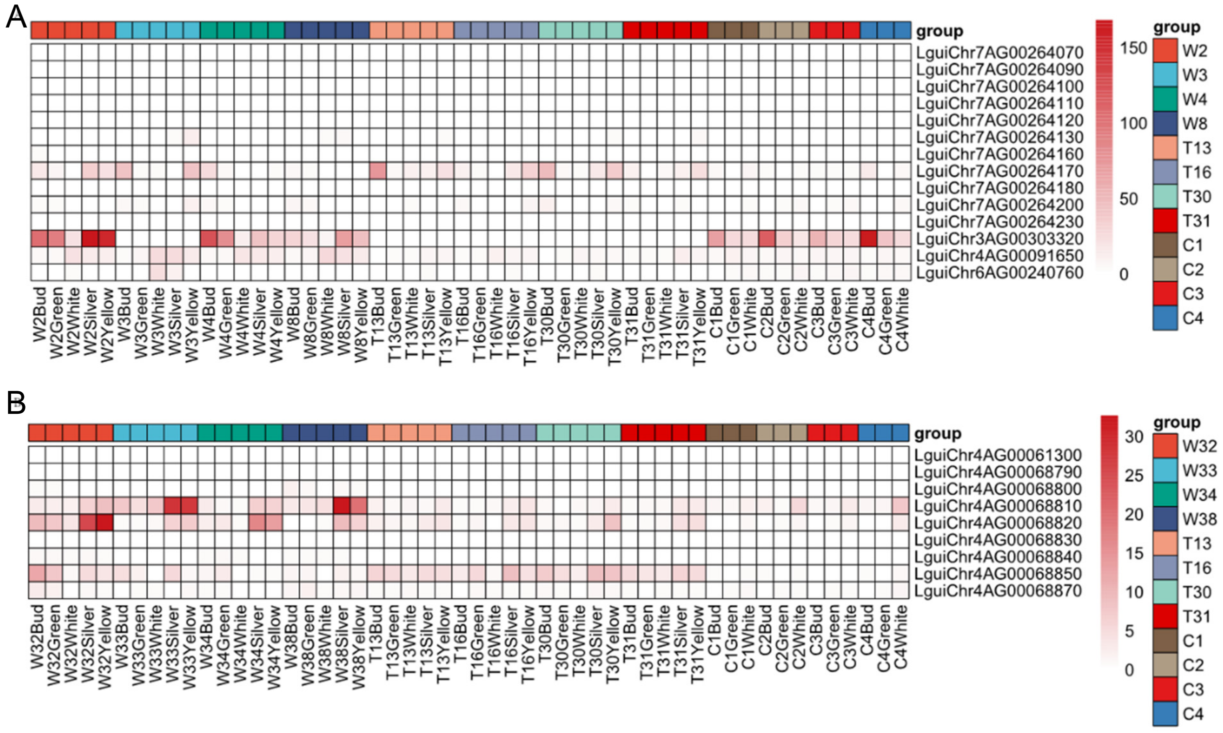

Figure S9 Expression of genes associated with GWAS signals for quality traits. **A:** Expression of genes associated with chlorogenic acid content. **B:** Expression of genes associated with dipsacoside B content. Heatmap colors represent z-score-normalized RNA-seq expression values (red, higher expression; blue, lower expression). The color scale ranges from －2 to 2 (z-score). Data were derived from flower buds at different developmental stages across cultivars W, T, and C.

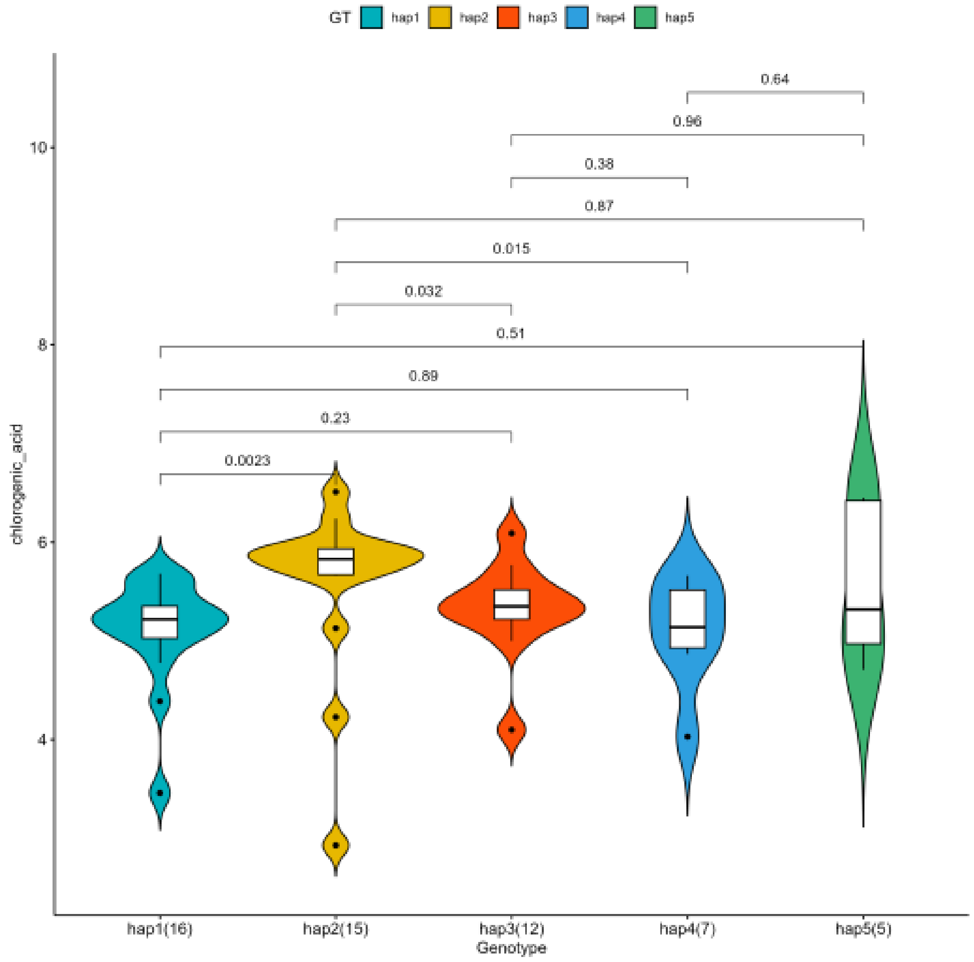

Figure S10 Haplotype analysis of LguiChr7AG00264170. For the haplotype analysis, the *n* values in the histogram and boxplots indicate the accession number with the corresponding haplotypes. In the boxplots, the 25% and 75% quartiles are shown as the lower and upper edges of boxes, respectively; the central lines denote the median and the small hollow squares indicate the mean. The whiskers extend to 1.5× the interquartile range and the small solid diamonds indicate outliers. The *P* values for two-sided Student's *t*-test are shown above the Boxplots.

A

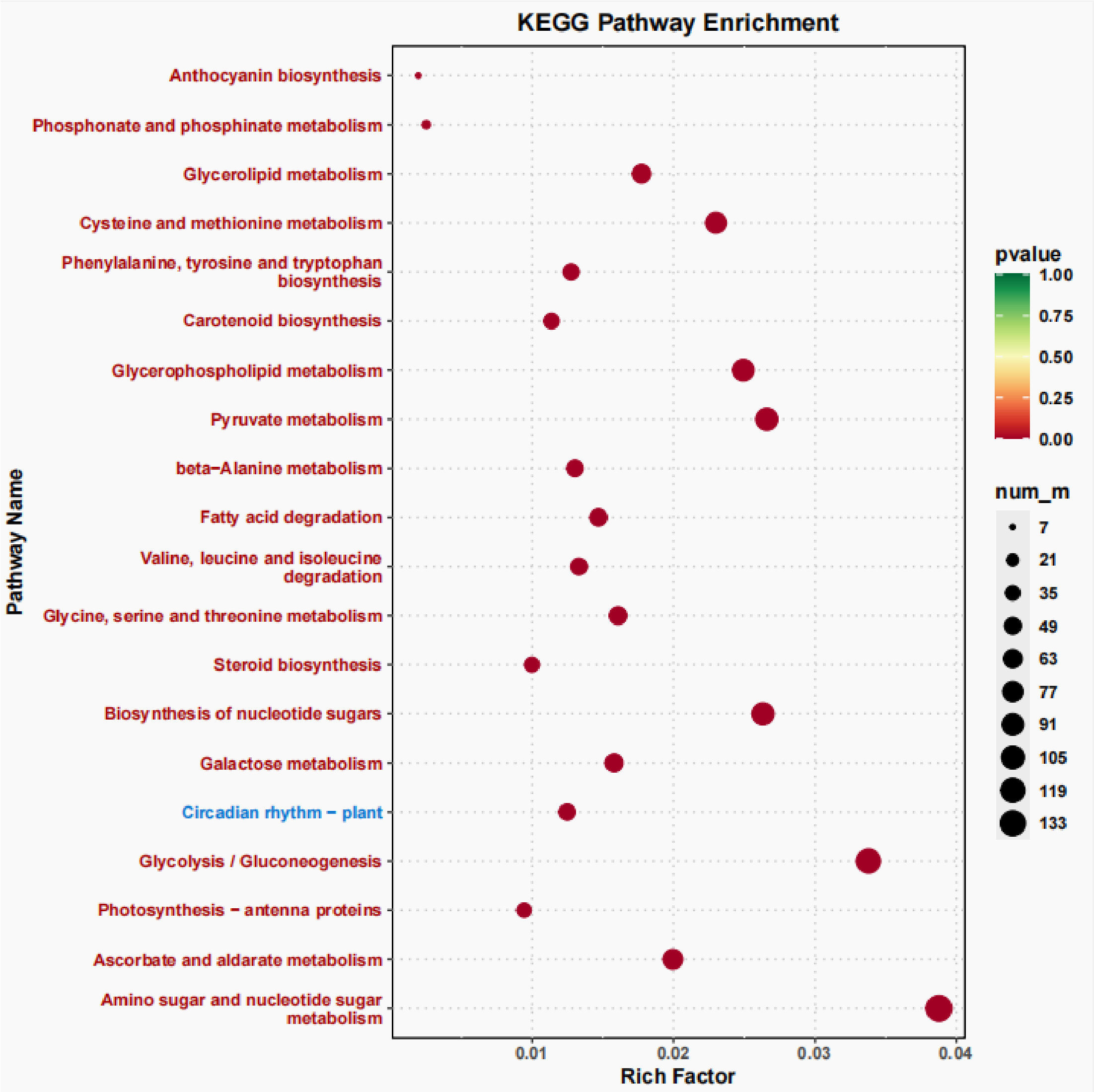
KEGG Pathway Enrichment
Anthocyanin biosynthesis
Phosphonate and phosphinate metabolism
Glycerolipid metabolism
Cysteine and methionine metabolism
Phenylalanine, tyrosine and tryptophan biosynthesis
Carotenoid biosynthesis
Glycerophospholipid metabolism
Pyruvate metabolism
beta−Alanine metabolism
Fatty acid degradation
Valine, leucine and isoleucine degradation
Glycine, serine and threonine metabolism
Steroid biosynthesis
Biosynthesis of nucleotide sugars
Galactose metabolism
Circadian rhythm − plant
Glycolysis / Gluconeogenesis
Photosynthesis − antenna proteins
Ascorbate and aldarate metabolism
Amino sugar and nucleotide sugar metabolism
Pathway Name
0.01
0.02
0.03
0.04
Rich Factor
pvalue
1.00
0.75
0.50
0.25
0.00
num_m
7
21
35
49
63
77
91
105
119
133

B

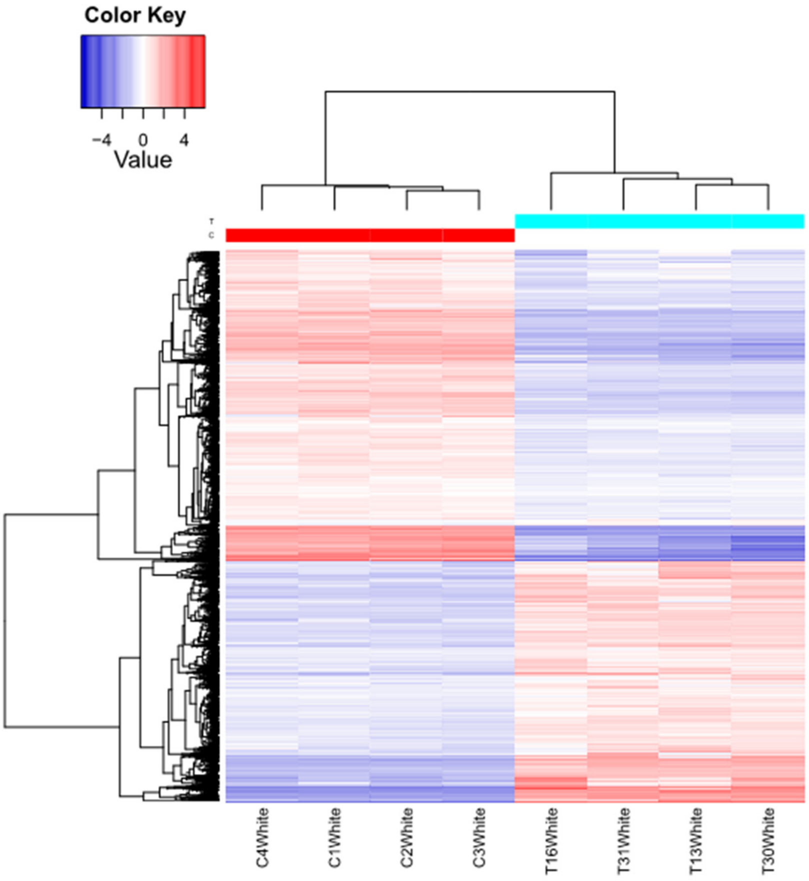

Figure S11 Combined analysis of transcriptomics and metabolomics in T-White vs. C-White and heat map of DEG. **A**: Co-enriched pathways from transcriptomics and metabolomics in T-White vs. C-White. **B**: The heat map of DEG between T-White and C-White shows very obvious expression differences, especially in the genes of the Pentose and glucuronate interconversions pathway.

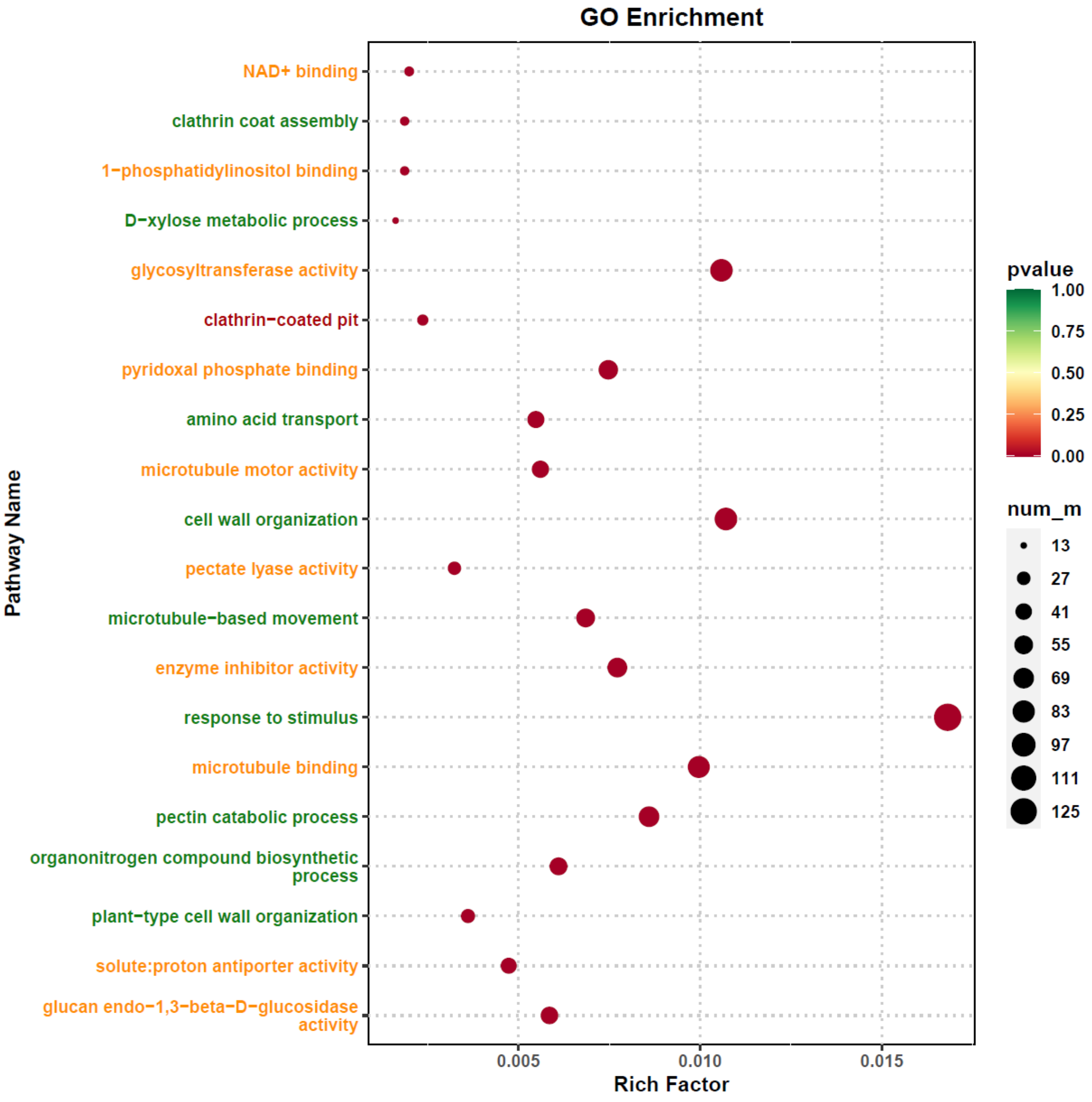

Figure S12 Combined analysis of transcriptomics and metabolomics in W-Green vs W-Silver.
